# Genome characterization and comparative genomics of *Limosilactobacillus reuteri* HDB isolated from the gut of an Indian infant

**DOI:** 10.3389/fmicb.2026.1780782

**Published:** 2026-04-17

**Authors:** Hemang D. Brahmbhatt, Priyank Chavda, Dhruvinkumar Vadee, Amrutlal K. Patel, Snehal Bagatharia

**Affiliations:** Department of Science and Technology, Gujarat Biotechnology Research Centre, Government of Gujarat, Gandhinagar, India

**Keywords:** comparative genomics, hybrid assembly, infant microbiome, *Limosilactobacillus reuteri*, pangenome analysis

## Abstract

*Limosilactobacillus reuteri*, formerly *Lactobacillus reuteri*, is a rod-shaped, Gram-positive, facultative anaerobe that colonizes the gastrointestinal tract of most vertebrates, including humans. We report the first isolation of *L. reuteri* strain HDB from the stool of a healthy Indian infant. Species assignment using the Type (Strain) Genome Server (TYGS) placed HDB within the *L. reuteri* clade, showing closest affinity to *L. reuteri* subspecies *porcinus* (dDDH 69.7%) yet clustering phylogenomically with *L. reuteri* DSM 17938. Hybrid *de novo* assembly (Illumina + Oxford Nanopore GridION MK1) generated a single circular 2,226,956 bp chromosome (GC 39.04%) encoding 2,160 CDS. Functional annotation identified genes involved in vitamin B_12_ biosynthesis, reuterin production, and probiotic functions, along with enriched carbohydrate and cofactor metabolic pathways. Comparative analysis with 59 *L. reuteri* genomes revealed a pangenome of 11,725 gene families, including 944 core gene families, 171 soft-core gene families, 1912 shell gene families, and 8698 cloud gene families, highlighting notable diversity. Core-genome phylogeny aligns HDB closely with the reference strain DSM 17938, confirming its identity as a human-associated lineage. dN/dS analysis indicated strong purifying selection across host niches, with no evidence of widespread positive selection. Genome-scale modeling predicts expanded carbohydrate flux in HDB against global references. The genetic background, along with its conserved metabolic features, suggests that HDB carries genomic characteristics commonly associated with human-derived *L. reuteri* strains. These observations support its consideration for further evaluation as a regionally sourced probiotic candidate. These conclusions are based on genomic and computational predictions and require experimental validation through adhesion, colonization, and safety studies.

## Introduction

Recent genome-based taxonomic revisions have reclassified *Lactobacillus reuteri* into the genus *Limosilactobacillus*. This change was driven by improved phylogenetic resolution obtained from whole-genome comparative analyses (Zheng et al., 2020). Although it shares several phenotypic characteristics with other lactic acid bacteria, comparative genomic analyses consistently position it on a distinct evolutionary branch within the Lactobacillaceae. This separation explains its lineage-specific divergence within the family (Frese et al., 2011; Walter et al., 2011). Ecologically, the species demonstrates notable versatility. It commonly inhabits the human gut, occurs across diverse vertebrate hosts including pigs, poultry, rodents, and in a range of fermented foods (Frese et al., 2011; Oh et al., 2010). Clinically, interest in *L. reuteri* has expanded over the past decade, with studies linking the species to digestive benefits (Bell et al., 2022; Gao et al., 2015; Hendrikx et al., 2019; Hu et al., 2017; Hunter et al., 2012; Jiang et al., 2016; Qi-Xiang et al., 2020), modulation of immune and autoimmune responses (He et al., 2017; Zegarra-Ruiz et al., 2019), and broader physiological effects, including bone health and neuro-gut interactions (Alghetaa et al., 2021; Chen et al., 2021; Chiang et al., 2021; Luo et al., 2018; McCabe et al., 2015; Natividad et al., 2018; Ni et al., 2017; Sgritta et al., 2019; Zhao et al., 2023). Its safety is well established; the U.S. FDA recognizes it as GRAS, and strains such as DSM 17938 are used routinely for managing infantile colic with demonstrated efficacy (Indrio et al., 2008; Savino et al., 2010).

The development and composition of the human gut microbiota are shaped by multiple interacting factors, like age, diet, host genetics, geography and general health status (Dubey et al., 2016; Kabeerdoss et al., 2013; Shetty et al., 2017). Colonization in early life, particularly during infancy, is now recognized as central to immune maturation, intestinal homeostasis and metabolic programing (Bäckhed et al., 2015; Yatsunenko et al., 2012). In the Indian context, large initiatives such as the LogMPIE pan-India study (Dubey et al., 2016) and several infant cohort investigations (Kabeerdoss et al., 2013) have expanded our understanding, yet most datasets still lack strain-level resolution and deeper functional annotation compared with major global microbiome efforts. Within this landscape, knowledge of *L. reuteri* in Indian populations remains limited. Much of the available literature relies on commercially sourced probiotic strains rather than strains isolated directly from Indian hosts, and indigenous human-derived isolates have rarely been characterized at the whole-genome or functional level. This gap is noteworthy given that strain-level diversity in *L. reuteri* is a well-documented example of adaptive radiation in the vertebrate gut, where long-term host association has produced distinct, host-specialized lineages with unique genetic and functional signatures (Frese et al., 2011; Oh et al., 2010; Walter et al., 2011). Consequently, isolating and fully characterizing *L. reuteri* strains from Indian infants or adults is essential for understanding host-specific adaptation and for evaluating their suitability in probiotic or therapeutic applications tailored to local needs. Accordingly, this study aimed to sequence and characterize *L. reuteri* HDB, an Indian infant isolate, to establish its phylogenomic position, functional capacity, and probiotic potential in relation to global *L. reuteri* diversity.

This study presents the first genome-level characterization of *L. reuteri* HDB, isolated from an Indian infant, and situates it within the wider comparative genomic landscape of the species. Using a hybrid sequencing strategy combining the accuracy of Illumina reads with the long-read continuity of Oxford Nanopore data, we generated a high-quality, complete genome. Functional annotation enabled us to map the major probiotic-linked pathways, including the full complement of genes required for cobalamin biosynthesis and the metabolic machinery for reuterin production. Comparative analysis with 59 *L. reuteri* genomes representing diverse hosts and regions provided insights into the evolutionary placement, host association and genomic variability of the HDB strain. By integrating hybrid assembly, pangenome structure, phylogenomics, selection analysis, and genome-scale metabolic reconstruction, we sought to define its genomic architecture and evaluate lineage-level conservation relative to established human-associated strains. Together, these results give us a clear picture of how *L. reuteri* adjusts to Indian infant gut conditions, and how strains like ours can be explored further for nutrition or health applications.

## Materials and methods

### Ethical approval and consent

A stool sample was collected from a 3-month-old breastfed Indian girl. Neither the girl nor her mother received any antibiotics in the recent past. Stool collection was conducted as per the study protocol approved by the Institutional Ethics Committee, Gujarat Biotechnology Research Centre (Approval No. GBRC/Ethics/15/Vitiligo/2023-24). Moreover, written consent from parents was taken before sample collection.

### Sample acquisition

Approximately 3-5 g of freshly voided stool was collected aseptically in a sterile container and immediately transferred to the laboratory on ice within 1 h of collection. Sample was aliquoted and stored at -80°C until further use.

### Bacterial isolation and taxonomic verification

For bacterial isolation, approximately 250 mg of stool sample was suspended in 1 mL sterile phosphate-buffered saline (PBS; pH 7.2) and vortexed thoroughly inside a Thermo Forma Anaerobic System (Model 1025) workstation (Thermo Fischer Scientific) and maintained under strict anaerobic conditions (85% N_2_, 10% H_2_, 5% CO_2_ with palladium catalyst). The suspension was serially diluted (10^– 2^–10^– 8^) and inoculated on selective media in serum bottles by roll bottle method, including Brain Heart Infusion (Hi-Media, India), Wilkins Chalgren anaerobic agar (TM media, India), Bifidobacterium specific anaerobic media (TM media, India) and Gifu anaerobic medium (TM media, India), supplemented with 0.05% L-cysteine (reductant) and 0.002% resazurin as a redox indicator. The media were pre-flushed with sterile CO_2_ gas to remove residual oxygen and to maintain anaerobic conditions during incubation. Serum bottles were incubated at 37°C for 72 h under anaerobic conditions. A total of four neonatal samples (two infant stool and two meconium samples) were processed under identical anaerobic culture conditions. More than 20 distinct bacterial isolates were obtained, and one isolate identified as *Limosilactobacillus reuteri* (strain HDB) was selected for whole-genome sequencing based on confirmed species identification and relevant genomic features. Distinct colonies were selected from all bottles, and sub-cultured them. Later, bacteria were subjected to multiple detection methods for identification.

For taxonomic verification, isolates were analyzed using matrix-assisted laser desorption/ionization time-of-flight mass spectrometry (MALDI-TOF MS; Autoflex maX, Bruker, Italy) following the manufacturer’s protocol. Spectra were acquired in linear mode (m/z 1,800-20,000 Da) and matched against the Biotyper Compass Explorer database (v4.1.100). Colonies presumptively identified as *Limosilactobacillus reuteri* were further confirmed by 16S rRNA gene sequencing. Genomic DNA was extracted using the QIAamp DNA Mini Kit (Qiagen, Germany), and the 16S rRNA gene was amplified with primers 27F (5′-AGAGTTTGATCCTGGCTCAG-3′) and 1429R (5′-GGTTACCTTGTTACGACTT-3′) using Emerald PCR Master Mix (Takara, Japan). Amplicons (∼1500 bp) were purified (ExoSAP-IT, Thermo Fisher, United States) and sequenced on an ABI 3500 XL Genetic Analyzer using BigDye Terminator v3.1 chemistry. Consensus sequences were assembled using CodonCode Aligner (v9.0) and the assembled sequence was submitted to BLASTn search against the NCBI GenBank database for preliminary species identification. For phylogenetic analysis, the 16S rRNA gene sequence of *L. reuteri* HDB was aligned with sequences of closely related type strains retrieved from the NCBI RefSeq database using MAFFT (v7.5). A Maximum Likelihood phylogenetic tree was constructed using IQ-TREE2 under the GTR + G substitution model with 1,000 ultrafast bootstrap replicates. *Limosilactobacillus fermentum* ATCC 14931 was used as the outgroup. Bootstrap support values ≥ 70% are shown at internal nodes. Taxonomic identification was further validated using the Type (Strain) Genome Server (TYGS) for genome-based taxonomic analysis. Digital DNA-DNA hybridization (dDDH) values and confidence intervals were calculated using the Genome-to-Genome Distance Calculator (GGDC) with formulas d0, d4, and d6. Phylogenomic analysis was performed using the Genome Blast Distance Phylogeny (GBDP) approach under algorithm “trimming” and distance formula d5. It is noted that the TYGS identification flagged remark [R1] for strain HDB, indicating that the genome of the closest type strain (*L. reuteri* DSM 20016) was not available in the TYGS database at the time of analysis; species identity was therefore confirmed by ANI analysis.

### DNA isolation and whole-genome sequencing

Genomic DNA was extracted using the CTAB method optimized for Gram-positive LAB, involving lysozyme lysis followed by CTAB precipitation to remove polysaccharides. This yielded high-MW DNA suitable for hybrid short- and long-read sequencing. DNA size was determined using a Qubit fluorometer (concentration) and 0.8% agarose gel electrophoresis (integrity). The library from 20 ng genomic DNA was prepared using the QIAseq FX DNA Library Kit as per the manufacturer’s protocol. For short-read sequencing, libraries were prepared using the QIAseq FX DNA Library Kit (Qiagen, Germany) following the manufacturer’s instructions. Sequencing was performed on the Illumina MiSeq platform (Illumina, United States) using a 300-cycle (2 × 150 bp) reagent kit on an SP flow cell, generating high-quality paired-end reads. For long-read sequencing, 400 ng genomic DNA was processed using the Oxford Nanopore Technologies (ONT) ligation sequencing gDNA-native barcoding kit (SQK-NBD114.96, version 14) and sequenced on a GridION Mk1 platform (ONT, United Kingdom) equipped with a FLO-MIN114 (R10.4.1) flow cell. Basecalling was performed using Guppy (v6.5.7) in high-accuracy mode. The hybrid sequencing strategy combining Illumina short reads and ONT long reads enabled high-quality *de novo* genome assembly and downstream comparative genomic analysis.

### Genome assembly and annotation

Raw Illumina reads were subjected to quality control using FastQC (v0.11.9), and adapter trimming was performed with Trimmomatic (v0.39) (Bolger et al., 2014). Reads with an average Phred quality score below 30 and a minimum length below 100 bp were discarded. For Nanopore data, raw reads were adapter-trimmed using Porechop (v0.2.4) (Bonenfant et al., 2022) to remove sequencing adapters and chimeric fragments, followed by quality and length filtering using Filtlong (v0.2.1), retaining reads longer than 1 kb. All bioinformatics analyses employed default parameters of the respective software versions unless explicitly stated.

Hybrid *de novo* assembly was carried out using Unicycler (v0.5.0) (Wick et al., 2017), which integrates short Illumina reads with long Oxford Nanopore reads to generate a complete, circularized genome. Assembly quality and completeness were assessed using QUAST (v5.2.0) (Gurevich et al., 2013) for contiguity metrics (N50, GC content, and total assembly size) and BUSCO (v5.7.1) (Manni et al., 2021) against the *Lactobacillales_odb10* lineage dataset to evaluate single-copy ortholog recovery.

Accurate taxonomic identification of the sequenced isolate is essential prior to downstream functional and comparative genomic analysis. Species identity was confirmed using ribosomal multilocus sequence typing (rMLST). The consensus genome assembly was analyzed against the rMLST genome database.^1^ rMLST assigns species with high sensitivity and specificity based on allelic profiles of 53 ribosomal protein subunit genes.

Structural and functional annotation of the assembled genome was performed using Prokka (v1.14.6) (Seemann, 2014), RAST (Aziz et al., 2008), and the Bacterial and Viral Bioinformatics Resource Center (BV-BRC) annotation pipeline (Olson et al., 2023). Gene prediction was refined using GeneMarkS-2 (Lomsadze et al., 2018), and predicted coding sequences (CDS), tRNAs, and rRNAs were categorized into functional subsystems. Comprehensive annotation included mapping of gene functions to the Cluster of Orthologous Groups (COG), Gene Ontology (GO), and KEGG metabolic pathway databases. The annotated genome was used for subsequent comparative genomic and phylogenetic analysis. Operon synteny was assessed by examining gene order and genomic coordinates within the assembled genome. Genomic regions containing the pdu and cob/cbi-hem loci were manually inspected to confirm contiguity and preserved gene organization based on annotated CDS positions in the final assembly.

### Comparative genomics and phylogenetic analysis

To investigate evolutionary relationships and host-specific diversity, the complete genome of *Limosilactobacillus reuteri* HDB was compared with 59 publicly available *L. reuteri* reference complete or high-quality genomes (Assembly status: Complete) retrieved from the NCBI database. Pairwise genomic similarity was calculated using FastANI (v1.33) to determine Average Nucleotide Identity (ANI) values. Core- and pan-genome analysis were performed with Roary (v3.13.0) using a 95% BLASTp identity cutoff, classifying genes into core (≥ 95% of strains), soft-core (99-95%), shell (95-15%), and cloud (< 15%) categories. A multiple sequence alignment of the core genome was generated within the Roary pipeline and used to construct a maximum-likelihood phylogenetic tree in IQ-TREE (v2.2.2) with 1000 ultrafast bootstrap replicates. The resulting tree was visualized and annotated using iTOL (v7.3) (Letunic and Bork, 2021). ANI matrices and core-genome clustering patterns were used to infer genetic relatedness among strains. The analysis revealed distinct host-specific clustering, with *L. reuteri* HDB forming a closely related clade with the reference strain *L. reuteri* DSM 17938, indicating high genomic similarity and potential functional conservation. Whole genome synteny between *L. reuteri* HDB and its closest relative *L. reuteri* DSM 17938 (99.94% ANI) was assessed using MUMmer 4.0.0beta2.^2^ Nucleotide sequences of both complete chromosomes were aligned using nucmer with default progressive Mauve-like parameters (-mum -maxmatch -c 500 -b 1000), followed by filtering of alignments shorter than 500 bp (delta-filter -minlength 500). Dot plots were generated using mummerplot (-fat -png 3,000 × 3,000 -layout) to visualize collinearity and identify potential structural rearrangements including inversions, translocations, or duplications. To investigate adaptive evolution specific to the human infant gut niche, pairwise Ka/Ks (dN/dS) ratios were calculated for 944 core genes comparing *L. reuteri* HDB and human-associated strains against host-specialized porcine (*n* = 10) and poultry (*n* = 7) lineages. Codon-aware pairwise alignments were generated for each core gene using the Roary core genome output, and Ka/Ks ratios were calculated using the Yang-Nielsen (YN00) method implemented in KaKs_Calculator 2.0. Only gene pairs with a minimum of five strain comparisons (N_pairs ≥ 5) were retained for downstream analysis. Genes were ranked by their mean Ka/Ks ratio to identify loci exhibiting the highest relative non-synonymous substitution rates in human-associated strains compared to porcine and poultry lineages. A Ka/Ks ratio < 1 indicates purifying selection, = 1 neutral evolution, and > 1 positive selection.

Prophage regions were identified using PHASTER (PHAge Search Tool Enhanced Release)^3^ by submitting the complete HDB genome sequence. Regions were classified as intact (score ≥ 90), questionable (70-90), or incomplete (< 70) based on PHASTER’s scoring algorithm. Insertion sequence (IS) elements were identified using ISfinder^4^ and cross-validated against Prokka annotation outputs. IS element positions were extracted from GFF annotation files and overlaid with MUMmer alignment breakpoint coordinates (gaps > 5 kb in nucmer alignments) to assess spatial association between IS elements and structural rearrangement sites.

### Functional annotation and pathway analysis

Functional annotation of the *L. reuteri* HDB genome was carried out using EggNOG-mapper v2.1.9 (Cantalapiedra et al., 2021) (local; DIAMOND engine, default parameters) and cross-validated with Galaxy’s EggNOG-mapper (eggNOG 5.0 database), assigning each CDS to Cluster of Orthologous Groups (COG) functional class. After the mapping, the distribution of genes across these categories was calculated and the profiles were plotted in GraphPad Prism (v9.0). Gene Ontology (GO) annotations were obtained from the same EggNOG output and grouped into Biological Process, Molecular Function, and Cellular Component categories through OmicsBox (v2.1.0) (Götz et al., 2008), providing an overview of broad functional trends. For metabolic reconstruction, the genome was processed through the RAST server and the BV-BRC pipeline, and the enzyme annotations from this step were linked to Kyoto Encyclopedia of Genes and Genomes (KEGG) pathways using the KEGG Automatic Annotation Server (KAAS) (Moriya et al., 2007). This allowed us to check pathway continuity and identify modules of interest. We examined the *pdu* operon involved in propanediol utilization and the full cobalamin biosynthesis pathway by confirming the presence of their constituent KEGG Orthologs. Taken together, these steps gave a clear picture of the functional capacities encoded in the HDB genome, spanning central metabolism, stress-related functions, and other features associated with probiotic performance. To assess the conservation of exopolysaccharide and levan biosynthesis loci across *L. reuteri* diversity, a comparative gene presence-absence analysis was performed across 60 *L. reuteri* genomes including HDB. Target loci comprising levS, gtfA_1, gtfA_2, galE, rmlB_1, ftsW, rodA, and murJ were queried against the Roary pangenome gene presence-absence matrix (95% BLASTp identity cutoff). Strains were grouped by host niche (human, porcine, poultry, rodent, other) and gene presence-absence patterns were visualized as a binary heatmap to evaluate host-associated conservation patterns.

### Genome-scale metabolic reconstruction and flux balance analysis

Genome-scale metabolic models (GEMs) for *L. reuteri* HDB and two global reference strains (*L. reuteri* JCM1112 and *L. reuteri* DSM 17938) were reconstructed from their complete genome sequences using the ModelSEED pipeline implemented within the KBase platform. Genome annotation was performed using the RASTtk pipeline prior to model reconstruction. Flux balance analysis (FBA) was conducted under identical complete-medium conditions with biomass production defined as the objective function, yielding a predicted growth rate (objective value) for each strain.

Pathway-level network composition was examined by extracting reactions annotated to carbohydrate and cofactor metabolic pathways based on KEGG pathway assignments embedded within each GEM. Comparative analysis of reaction representation across carbohydrate metabolism (glycolysis/gluconeogenesis, TCA cycle, pentose phosphate pathway, and pyruvate metabolism) and selected cofactor biosynthesis pathways (porphyrin, vitamin B6, ubiquinone, riboflavin, and thiamine biosynthesis) was performed to assess structural similarities and subtle network-level differences between HDB and global reference strains.

### Secondary metabolite biosynthesis, probiotic, and safety-related gene prediction

To examine secondary metabolite pathways and safety-related features in the HDB genome, we relied on a set of commonly used bioinformatic tools. AntiSMASH (v7.0) (Blin et al., 2021) and gutSMASH (v2.0.1) (Palladino et al., 2022) were used initially to survey biosynthetic clusters, with particular attention to the *pdu-cob-hem* region that links glycerol metabolism to reuterin formation. Possible bacteriocin loci were then reviewed using BAGEL5 (v2.0) (van Heel et al., 2018), and outputs from ProbioMine were manually checked to identify genes associated with adhesion (*mapA, mub, ef-tu, gap*), stress response (*groEL, clpL, dnaK*), exopolysaccharide synthesis, and bile salt hydrolase activity. For the safety component, AMR determinants were screened through the CARD database (v3.3.2) (Alcock et al., 2020) and Resistance Gene Identifier (RGI) tool (v6.0.3) (Feldgarden et al., 2019), while virulence-related genes were examined against the VFDB 2024 release (Chen et al., 2005). We also ran PlasmidFinder (v2.1) (Carattoli et al., 2014) to identify any plasmid replicons or plasmid-linked elements, and CRISPRCasFinder (v4.2.20) (Couvin et al., 2018) was used to document CRISPR arrays. Across these analyses, we did not detect virulence factors, pathogenicity markers, transferable AMR genes, or plasmid-borne risks. In contrast, the presence of adhesion-, stress-, and antimicrobial-associated genes points to a genomic profile that is compatible with probiotic use.

### Statistics

Data visualization was performed using GraphPad Prism v9.0.

## Results

### Isolation and Identification of *Limosilactobacillus reuteri* HDB

**FIGURE 1:**
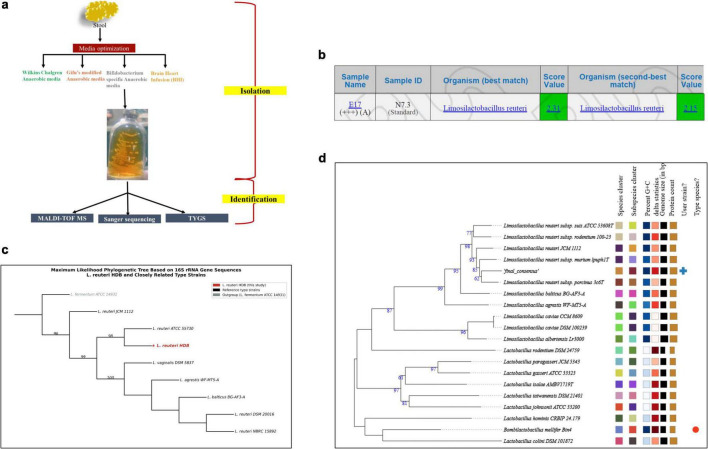
Isolation and taxonomic identification of *Limosilactobacillus reuteri* HDB. **(a)** Workflow outlining isolation from infant stool under anaerobic conditions following media optimization using modified Gifu Anaerobic Medium (GAM), Bifidobacterium-specific medium, and Brain Heart Infusion (BHI) agar. Colonies were purified and subjected to molecular and proteomic identification. **(b)** MALDI-TOF MS analysis (Bruker system) showing log-score values > 2.0, confirming species-level identification as *L. reuteri*. **(c)** Maximum Likelihood phylogenetic tree based on a 1,465 bp 16S rRNA gene sequence generated by Sanger sequencing. Sequences of closely related type strains were retrieved from NCBI RefSeq and aligned using MAFFT (v7). The tree was constructed in IQ-TREE2 under the GTR + G substitution model with 1,000 ultrafast bootstrap replicates; bootstrap values ≥ 70% are shown at nodes. *Limosilactobacillus fermentum* ATCC 14931 was used as the outgroup. **(d)** Whole-genome phylogenomic placement using the Type (Strain) Genome Server (TYGS), based on Genome BLAST Distance Phylogeny (GBDP). Bootstrap values (100 replicates) are indicated, confirming clustering within the *L. reuteri* species complex.

Anaerobic cultivation of the infant stool sample on selective and anaerobe-supporting media yielded morphologically distinct colonies after 48–72 h at 37°C. Growth was strongest on Bifidobacterium-specific anaerobic medium supplemented with L-cysteine and the redox indicator resazurin, consistent with effective oxygen scavenging under CO_2_-enriched conditions. Small, smooth, creamy white colonies were picked and serially re-streaked to purity (Figure 1a). MALDI-TOF MS analysis of the purified isolate generated mass spectra with high-quality profiles, yielding a log score of 2.31 against the Biotyper database, providing reliable species-level identification as *Limosilactobacillus reuteri* (Figure 1b). Confirmatory identification by 16S rRNA gene sequencing generated a 1,465 bp consensus sequence showing 99.6% identity to the reference strain *L. reuteri* DSM 20016 (GenBank accession NR_075036). Maximum Likelihood phylogenetic analysis placed *L. reuteri* HDB within a well-supported subclade (bootstrap = 98%) with *L. reuteri* ATCC 55730, clearly distinct from other *Limosilactobacillus* species, confirming species identity (Figure 1c). BLAST similarity results are provided in Supplementary Figure 1. TYGS analysis confirmed this classification, with highest dDDH values against *Limosilactobacillus reuteri* subsp. porcinus 3c6T (69.7% ± 2.9%) and *Limosilactobacillus reuteri* JCM 1112 (65.0% ± 2.8%), and GC content differences < 1%. The GBDP-based phylogenomic tree placed HDB robustly within the *L. reuteri* cluster with strong bootstrap support, corroborating both 16S and MALDI-TOF results (Figure 1d).

### Genome sequencing, hybrid assembly, and quality assessment

**FIGURE 2:**
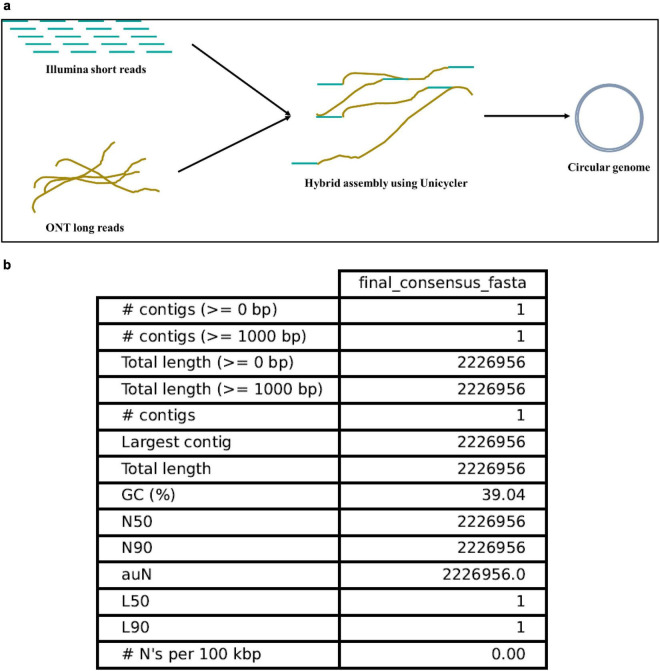
Hybrid genome sequencing and assembly of *L. reuteri* HDB. **(a)** Schematic of hybrid assembly strategy integrating Illumina short-read sequencing (paired-end, quality filtered using FastQC and Trimmomatic) and Oxford Nanopore long-read sequencing (adapter trimming with Porechop and filtering using Filtlong). Hybrid *de novo* assembly was performed using Unicycler (v0.5.0). **(b)** Assembly quality metrics generated by QUAST (v5.2.0), indicating a single circular chromosome (2,226,956 bp), GC content of 39.04%, and N50 equal to total genome length.

A hybrid sequencing strategy was used to maximize contiguity and base-level accuracy (Figure 2a). Illumina MiSeq generated 0.919 million paired-end reads (2 × 150 bp) totaling 0.276 Gb, of which 94.5% exceeded Q30 (post filtering). Oxford Nanopore Technologies (ONT; GridION, FLO-MIN114, ligation-based native barcoding v14) produced 887,053 bp raw long reads with a read length N50 of 1.30 kb and mean Phred-equivalent quality of 14.7. After adapter trimming (Porechop) and quality/length filtering (Filtlong; > 1 kb), ∼25% of ONT reads (prioritizing the highest quality and longest reads) were retained for assembly. Post-QC Illumina and ONT datasets provided estimated mean coverages of 104x and 100x, respectively (assuming a ∼2.23 Mb genome).

Hybrid *de novo* assembly with Unicycler yielded a single, circular chromosome of 2,226,956 bp (GC 39.04%). QUAST reported an N50 equal to the full genome length with one contig and no ambiguous bases. Short-read mapping showed a high proportion of aligned Illumina reads with uniform coverage depth, indicating even sequencing and minimal collapsed repeats. Long-read mapping corroborated global contiguity and near-complete coverage of the assembly (Figure 2b).

Genome completeness assessed by BUSCO against the *lactobacillales_odb10* database (*n* = 402) revealed 99.5% complete BUSCOs (399 single-copy, 1 duplicated), 0.0% fragmented, and 0.5% missing genes, confirming a high-quality, complete assembly (Table 1). No extrachromosomal contigs were recovered in the final assembly. Plasmid replicon screening (PlasmidFinder) did not detect known plasmid backbones, suggesting a plasmid-free architecture for HDB under the growth conditions tested. The complete, plasmid-free circular genome provides a robust foundation for downstream comparative and functional analysis.

**TABLE 1:** Genome assembly quality and completeness of *L. reuteri* HDB.

Category	Parameter	Value
Assembly statistics	Genome size	2,226,956 bp
	Number of contigs	1
	Assembly N50	2 Mb
	Percent gaps	0.00%
	Complete BUSCOs	400 (99.5%)
	Single-copy	399 (99.3%)
BUSCO completeness (lactobacillales_odb10; *n* = 402)	Duplicated	1 (0.2%)
	Fragmented BUSCOs	0 (0.0%)
	Missing BUSCOs	2 (0.5%)

Assembly statistics of the complete genome of *Limosilactobacillus reuteri* HDB generated using hybrid Illumina and Oxford Nanopore sequencing. Assembly metrics were calculated using QUAST. Genome completeness was assessed using BUSCO v5 with the lactobacillales_odb10 dataset (n = 402 conserved single-copy orthologs).

The rMLST analysis unequivocally identified the assembled genome as *Limosilactobacillus reuteri* with 100% assignment confidence. Forty-nine out of fifty-three rMLST loci showed exact sequence identity to *L. reuteri* in the database, providing robust taxonomic validation for the newly sequenced strain. The rMLST database links for each locus confirmed highest similarity to deposited *L. reuteri* genomes, with cross-references to > 300 public genomes for some alleles (Table 2 and Supplementary Figure 2).

**TABLE 2:** rMLST-based species assignment.

Parameter	Value
Predicted species	Limosilactobacillus reuteri
Taxonomic support	100%
Full taxonomy	Bacillota > Bacilli > Lactobacillales > Lactobacillaceae > *Limosilactobacillus* > *Limosilactobacillus reuteri*
Total rMLST loci queried	53
Exact locus matches	49
Top database match	*L. reuteri* (rMLST genome database; *n* = 336)
Input file	final_consensus. fasta

Ribosomal multilocus sequence typing (rMLST) analysis performed using the PubMLST database. Fifty-three ribosomal protein loci were queried against the rMLST genome database to determine species-level assignment and taxonomic support.

### Genome annotation and functional landscape

**FIGURE 3:**
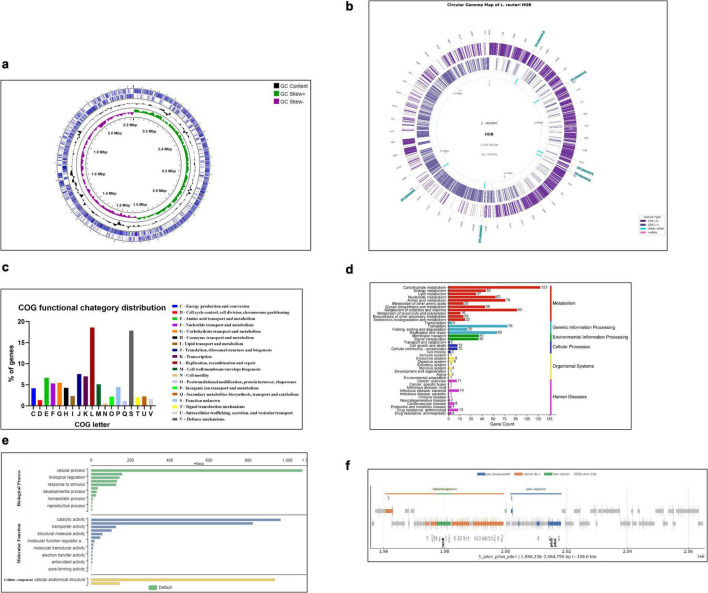
Structural and functional genomic features of *Limosilactobacillus reuteri* HDB. **(a)** GC content and GC skew distribution across the circular chromosome of *L. reuteri* HDB, calculated from the final hybrid assembly (Unicycler v0.5.0). GC skew (G-C/G + C) highlights replication origin and terminus symmetry across the 2.23 Mb genome. **(b)** Circular genome map generated from Prokka (v1.14.6) annotation, displaying coding sequences (CDS) on forward and reverse strands, rRNA/tRNA loci, GC content, and GC skew. The complete genome consists of a single circular chromosome (2,226,956 bp; GC 39.04%) without plasmids. **(c)** Cluster of Orthologous Groups (COG) classification was performed using EggNOG-mapper v2.1.9 with DIAMOND search (default settings). Genes were assigned to different COG categories. Higher representation was observed in replication/recombination/repair (L), carbohydrate metabolism (G), transcription (K), and translation (J), which is typical for lactic acid bacterial genomes. **(d)** KEGG pathway mapping was carried out using enzyme annotations from RAST and BV-BRC, followed by ortholog assignment through KAAS. Most genes were linked to metabolic pathways, particularly carbohydrate, amino acid, and cofactor metabolism, supporting the fermentative and biosynthetic nature of the strain. **(e)** Gene Ontology classification was generated using OmicsBox (v2.1.0) under Biological Process, Molecular Function, and Cellular Component categories. Catalytic activity and transporter activity were among the most represented terms, indicating active metabolism and substrate transport capacity. **(f)** Operon-level organization of the ∼108 kb pdu-cob/cbi-hem region involved in glycerol/1,2-propanediol utilization and cobalamin (vitamin B_12_) biosynthesis is shown. Gene order and continuity were manually verified from annotated CDS coordinates, confirming an intact and conserved cluster associated with reuterin-related metabolism.

Analysis of the *L. reuteri* HDB genome revealed an average GC content of 39.04%, consistent with reported values for *L. reuteri* species. GC skew analysis (Figure 3a) displayed distinct positive and negative regions indicative of leading and lagging strand biases, with transitions suggesting the probable locations of the origin and terminus of replication. The circular genome map generated by PROKKA annotation explains the distribution of 2,160 protein-coding sequences (CDS), 69 tRNA genes, and 18 rRNA operons across the *L. reuteri* HDB chromosome, with locus tags and gene labels emphasizing both functional and numerous hypothetical proteins (Figure 3b). The visualization reveals high gene density and homogenous distribution with multiple ribosomal RNA clusters (16S rRNA, 23S rRNA), essential metabolic capabilities (sugar metabolism, vitamin synthesis and amino acid metabolism) and abundant uncharacterized genes (hypothetical proteins). EggNOG-mapper assigned 2010 (93.1%) of CDS to orthologous groups, enabling COG-level functional classification. The most represented COG categories were L (Replication, recombination and repair) 18.6%, J (Translation, ribosomal structure and biogenesis) 7.50%, K (Transcription) 7.01%, and E (Amino acid transport and metabolism) 6.66%, consistent with a niche-adapted fermentative lifestyle reliant on carbohydrate uptake and processing (Figure 3c; descriptive distribution, no statistical comparisons performed). These genes abundance reflecting baseline metabolic and translational capacity typical of compact lactic-acid-bacteria genomes.

Functional annotation of the *L. reuteri* HDB genome based on KEGG pathways (Figure 3d) revealed a predominance of genes involved in metabolism, with the highest gene counts observed in carbohydrate, amino acid, and nucleotide metabolism. Substantial representation was also seen in genetic information processing pathways, such as transcription and replication, whereas relatively few genes were linked to cellular processes, organismal systems, and human disease pathways. Folate and cobalamin (vitamin B_12_) biosynthetic modules were well represented. Gene Ontology (GO) terms were distributed across Biological Process, Molecular Function, and Cellular Component (9 total terms), with enrichment in catalytic activity and transmembrane transporter activity, reflecting rich repertoires for carbohydrate uptake, stress protection, and metabolite interconversion (Figure 3e).

Particularly, the *pdu-cob-hem* gene cluster central to glycerol/1,2-propanediol metabolism and reuterin (3-hydroxypropionaldehyde) production was complete, including pdu genes and cbi/cob/hem operons. Associated cofactor biosynthesis genes (e.g., cob/cbi for cobalamin) were contiguous and displayed preserved gene order within a ∼108 kb genomic region, consistent with operon-level organization required for reuterin biosynthesis (Figure 3f). Additional loci putatively linked to probiotic function were identified, including exopolysaccharide (EPS) biosynthesis genes, stress chaperones (*groEL, dnaK, clpC*), and bile salt hydrolase (*bsh*), supporting physiological resilience in the gastrointestinal environment (Table 3).

**TABLE 3:** Key probiotic-associated and metabolic genes.

Gene	Function	Length (bP)	Product
*pduC*	Glycerol dehydratase (glycerol—3-hydroxypropionaldehyde)	1,602	Glycerol/diol dehydratase, large subunit
*pduD*	Dehydratase enzyme	1,140	Glycerol/diol dehydratase, medium subunit
*pduE*	Dehydratase enzyme	870	Glycerol/diol dehydratase, small subunit
*pduP*	Aldehyde dehy drogenase (detoxification/conversion of aldehydes)	1,449	Propionaldehyde dehydrogenase
*pduQ*	Dehydrogenase (redox enzyme)	1,236	1,3-propanediol dehydrogenase
*cobA*	Cobalamin (vitamin B_12_) biosynthesis enzyme	810	Uroporphyrinogen-III methyltransferase
*cbiT*	Cobalamin (vitamin B_12_) biosynthesis enzyme	960	Cobalamin biosynthesis protein
*cob*	Regulates cobalamin synthesis and energy metabolism via acetylation control	699	NAD-dependent protein deacetylase
*Igt*	Lipoprotein maturation	828	Phosphatidylglycerol–prolipoprotein diacylglyceryl transferase
*pgsA*	Involved in phospholipid biosynthesis	588	CDP-diacylglycerol–glycerol-3-phosphate 3-phosphatidyItransferase
*plsC*	Membrane lipid synthesis	732	1-acyl-sn-glycerol-3-phosphate acyltransferase
*tarF*	Cell wall teichoic acid biosynthesis	1,167	Teichoic acid glycerol-phosphate transferase
*cpoA*	Alpha-galactosyl-diacylglycerol synthesis; membrane lipid component	1,029	Alpha-galactosylglucosyldiacylglycerol synthase
*Dagk*	Maintains lipid homeostasis	1,014	Diacylglycerol kinase
*czcD*	Mediates cadmium, cobalt, and zinc export; detoxification	921	Cadmium, cobalt and zinc/H(+)-K(+) antiporter
*mprF*	Confers cationic antimicrobial peptide resistance	2,586	Phosphatidylglycerol lysyltransferase
*hemH*	Final step in heme biosynthesis; inserts Fe^2+^” into protoporphyrin IX	933	Ferrochelatase
*cobC*_1	B_12_ salvage and remodeling enzyme	654	Adenosylcobalamin/alpha-ribazole phosphatase
*rtpR*	B_12_-dependent DNA precursor synthesis	2,205	Adenosyleobalamin-dependent ribonucleoside-triphosphate reductase
*cobT*	Links nucleotide loop and lower ligand in B_12_ synthesis	1,053	Nicotinate-nucleotide–dimethylbenzimidazole phosphoribosyltransferase
*cobC*_2	Redundant copy involved in B_12_ activation	591	Adenosylcobalamin/alpha-ribazole phosphatase
*cobS*	Assembles nucleotide loop in cobalamin	762	Adenosylcobinamide-GDP ribazoletransferase
*cobU*	key B_12_ assembly enzyme	591	Bifunctional adenosylcobalamin biosynthesis protein
*hemL*	Heme biosynthesis enzyme	1,296	Glutamate-1-semialdehyde 2,1-aminomutase
*hemB*	Heme biosynthesis enzyme	972	Delta-aminolevulinic acid dehydratase
*hemC*	Heme biosynthesis enzyme	918	Porphobilinogen deaminase
*hemA*_1	Heme biosynthesis enzyme	1,266	Glutamyl-tRNA reductase
*cobQ*	Amidation in cobalamin pathway	1,512	Cobyric acid synthase
*cbiO*	Cobalt ion transport into cytoplasm	810	Cobalt import ATP-binding protein CbiO
*cbiQ*	Membrane component of cobalt ABC transporter	678	Cobalt transport protein CbiQ
*cbiN*	Forms channel for cobalt uptake	324	Cobalt transport protein CbiN
*chiM*	Transmembrane cobalt ion transporter	744	Cobalt transport protein CbiM
*cbiL*	Precorrin-2 C (20)-methyltransferase	699	Cobalt-precorrin-2 C (20)-methyltransferase
*Cbik*	Inserts cobalt into sirohydrochlorin during corrinoid formation	717	Sirohydrochlorin cobaltochelatase
*cbiJ*	Reduces precorrin intermediates in B_12_ synthesis	759	Cobalt-precorin-6A reductase

Selected genes associated with propanediol utilization (pdu operon), cobalamin (vitamin B_12_) biosynthesis, heme biosynthesis, lipid metabolism, antimicrobial resistance-related mechanisms, and stress adaptation identified from PROKKA-annotated genome and KEGG pathway mapping.

### Genomic safety profile and probiotic-relevant traits

To evaluate genomic safety, we screened HDB against AMR and virulence databases. No acquired antimicrobial resistance determinants were detected using CARD/ResFinder/AMRFinderPlus, and no virulence-associated loci were identified in VFDB/VirulenceFinder. A single cold shock protein gene (*cspA*) was flagged by PHI-base with 83.08% identity and 97.01% coverage, consistent with canonical stress response rather than virulence. Notably, PlasmidFinder reported no plasmid replicons, decreasing the likelihood of mobile, horizontally transferable resistance/virulence cargo. Collectively, these observations support a chromosomally encoded, low-risk safety profile (Tables 4A,B).

**TABLE 4A:** Genomic safety screening results.

Screening category	Antibiotic resistance genes (ARGs)			Virulence factors (VFs)			Pathogenicity genes	
Database	CARD	ResFinder	AMRFinder Plus	VFDB	Virulence Finder	PHL-base	Plasmid Finder	Probio Mine
Our isolate: *L. reuteri* HDB	Not detected	Not detected	Not detected	Not detected	Not detected	1 hit (see Table 4B)	Not detected	1

“Not detected” indicates no hits above database-specific thresholds. PPRS was calculated using ProbioMine; scores ≤ 4 indicate low-risk strains. *In silico* safety assessment of L. reuteri HDB using CARD, ResFinder, AMRFinderPlus (antibiotic resistance genes), VFDB and VirulenceFinder (virulence factors), PHI-base (pathogenicity-related genes), PlasmidFinder (plasmids), and ProBioMine (probiotic risk scoring). Default database thresholds were applied.

**TABLE 4B:** (PHI-base hit–CspA).

Isolate	Protein ID	Gene ID	Gene name	Identity	Coverge	*e*-value	Function
*L. reuteri* HDB	P0A355	CAA62903	CspA	83.08	97.01	1.96e–35	Cold shock protein

The single hit (CspA, P0A355) corresponds to a canonical cold shock protein involved in stress response and is not associated with virulence or pathogenicity. PHI-base screening result showing the single hit identified in the HDB genome. Identity, coverage, and *E*-value thresholds were based on BLASTp search against PHI-base curated entries.

We next inspected canonical probiotic trait loci. Genomic analysis revealed key probiotic-associated loci including stress tolerance modules comprising molecular chaperones (groEL, dnaK) and ATP-dependent Clp proteases (clpC, clpX, clpE), supporting gastrointestinal persistence. Bile adaptation was confirmed by the presence of bile salt hydrolase (bsh/cbh). Elongation factor Tu (tuf), a known moonlighting adhesin with surface-exposed epitopes, was identified, though the canonical mucus-binding protein (mub) was absent, *L. reuteri* HDB harbors alternative adhesion mechanisms including moonlighting adhesins GAPDH (*gap*) and enolase (*eno2*), both well-documented for surface exposure and mucin-binding activity in lactobacilli (Table 5). Analysis of the *L. reuteri* HDB genome identified a minimal Wzx/Wzy-independent EPS biosynthesis module consisting of two GtfA glycosyltransferases (*gtfa_1, gtfa_2*), UDP-glucose 4-epimerase (gale), dTDP-glucose 4,6-dehydratase (*rmlB*), peptidoglycan glycosyltransferases (*roda, ftsw*), and a GH68 levansucrase (*levS*). These loci (Table 6) suggest a predominant sucrase-type levan pathway complemented by core precursor enzymes and a MurJ-like flippase for glycan export, supporting biofilm formation and mucosal adhesion via EPS. The Probiotic Potential Risk Score (PPRS) from ProbioMine was 1 (low-risk), further supporting genomic suitability for probiotic development (Tables 4A,B). While genomic inference does not substitute for phenotypic validation, the combination of absence of concerning safety genes and presence of canonical probiotic traits provides a strong rationale for further functional testing.

**TABLE 5:** Adhesion and stress response genes.

Gene	Function	Length (bP)	Product
*ef-tu (tuf)*	A surface-exposed adhesin mediating mucin and epithelial attachment	1,191	Elongation factor Tu
*ef-ts (tsf)*	Supports protein synthesis under stress	876	Elongation factor Ts
*gap*	Moonlighting adhesin binding mucins and ECM components	1,008	Glyceraldehyde-3-phosphate dehydrogenase (GAPDH)
*eno2_1*	Binds plasminogen and mucins to promote adhesion	258	Enolase (isoform 1)
*eno2_2*	Isoform with similar glycolytic and surface-adhesion functions	1,080	Enolase (isoform 2)
*groEl*	Molecular chaperone that assists protein folding and protects from thermal and oxidative stress	1,629	60 kDa Chaperonin
*dnaK*	Stress-response chaperone preventing protein aggregation under heat or pH stress	1,866	Chaperone protein Dnak
*clpC*	Crucial for stress tolerance and protein homeostasis	2,238–2,493	ATP-dependent Clp proteases
*bsh*	Enhancing bile resistance and intestinal survival	609	Choloylglycine hydrolase

Genes associated with adhesion, mucin binding, bile tolerance, heat stress response, and protein folding identified from genome annotation and literature-curated probiotic markers.

**TABLE 6:** Wzx/Wzy-independent EPS biosynthesis module present in *L. reuteri* HDB genome.

Gene	Function	Length (bP)	Product
*gifa_1*	Glucansucrase activity	1542	UDP-n-acetylglucosamine–peptide n-acetylglucosaminyltransferase gtfa subunit
*gtfa_2*	Involved in levan or dextran synthesis	1503	UDP-n-acetylglucosamine–peptide n-acetylglucosaminyltransferase gtfa subunit
*gale*	Providing sugar precursors for EPS	996	UDP-glucose 4-epimerase
*roda*	Involved in peptidoglycan synthesis	1194	Peptidoglycan glycosyltransferase roda
*ftsw*	A lipid II flippase-like glycosyltransferase aiding cell wall and EPS export	1224	Putative peptidoglycan glycosyltransferase ftsw
*levs*	Contributes to levan-type EPS formation	1776	Levansucrase
*rmlb_1*	A precursor in rhamnose biosynthesis for EPS	1041	dTDP-glucose 4, 6-dehydratase
*murj*	Essential for polysaccharide assembly	1650	Lipid ii flippase murj

This table provides list of exopolysaccharide (EPS) and levan biosynthesis genes and their functions based on annotation and orthology analysis. Gene functions were assigned using PROKKA and KEGG annotations.

### Secondary metabolite and bacteriocin potential

**FIGURE 4:**
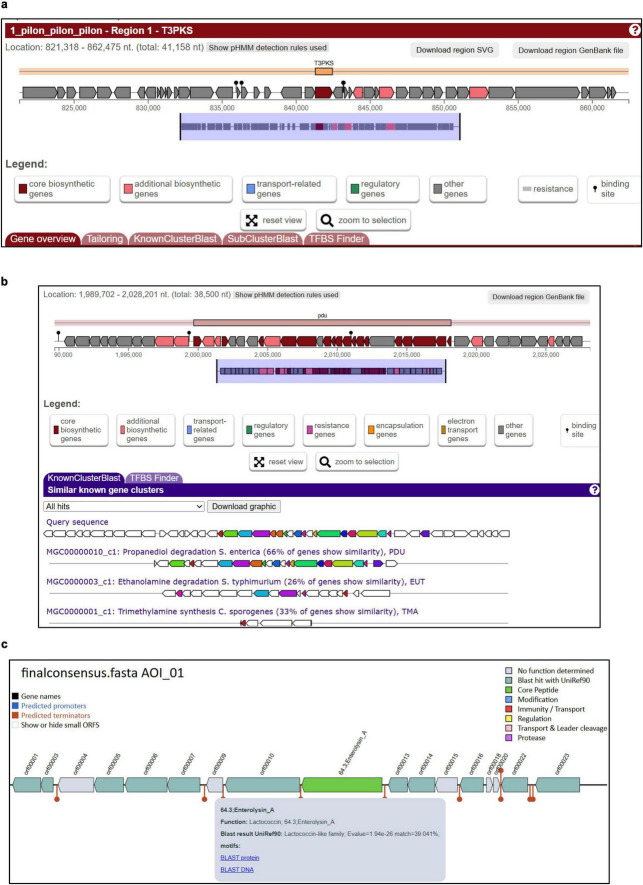
Specialized metabolic and antimicrobial gene clusters identified in *Limosilactobacillus reuteri* HDB. **(a)** Secondary metabolite gene clusters predicted using antiSMASH (v7.0, default bacterial settings). A Type III Polyketide Synthase (T3PKS) cluster (∼41 kb) was identified, containing core biosynthetic and associated genes. Cluster boundaries were defined based on antiSMASH detection rules. **(b)** Propanediol utilization (pdu) gene cluster identified using gutSMASH (v2.0.1), spanning ∼38.5 kb. The cluster contains core pdu genes along with associated regulatory and structural elements. KnownClusterBlast analysis showed similarity to previously reported propanediol metabolism loci. **(c)** The figure bacteriocin cluster detected using BAGEL (v4), corresponding to Enterolysin A like genes. Annotation was cross-checked with UniRef90 and conserved domain databases. Predicted structural, immunity, and transport genes are shown, suggesting potential antimicrobial function.

AntiSMASH detected one biosynthetic gene cluster (T3PKS) (Figure 4a). While gutSMASH detected two biosynthetic gene clusters. The most prominent was the complete *pdu-cob-hem* super-cluster, genetically equipping HDB to produce reuterin, a broad-spectrum antimicrobial. The cluster spanned 38.5 kb and included structural, regulatory, and transport components necessary for glycerol conversion and cofactor provision (Figure 4b). Bacteriocin analysis with BAGEL4 identified one bacteriocin-encoding region, that is Enterolycin A (Figure 4c). The presence of multiple antimicrobial systems reuterin and bacteriocin, indicates multipronged antagonism that could inhibit competing/pathogenic microbes within the gastrointestinal niche.

CRISPRCasFinder analysis revealed the complete absence of CRISPR arrays and associated Cas proteins in the *L. reuteri* HDB genome, indicating this strain lacks CRISPR-Cas adaptive immunity systems. The absence of this phage defense mechanism suggests reliance on alternative innate immunity strategies for survival in the dynamic gut environment.

### Comparative genomics: ANI, pangenome structure, and phylogeny

**FIGURE 5:**
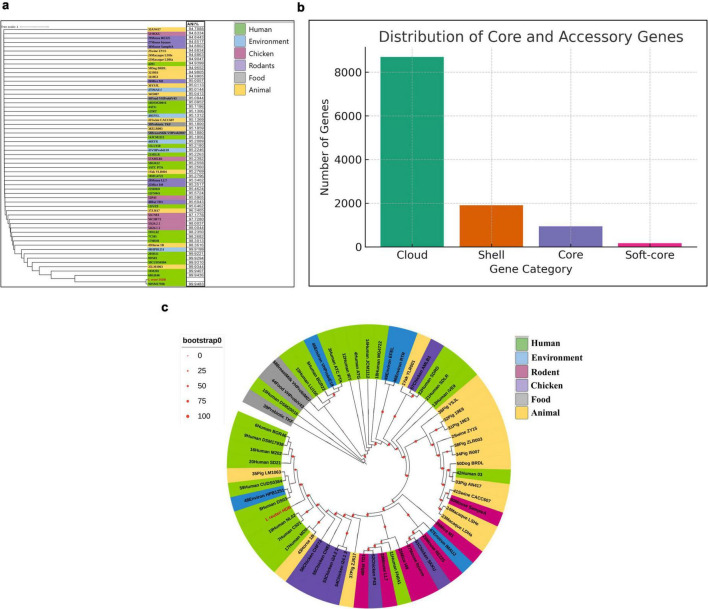
Comparative genomic analysis of *Limosilactobacillus reuteri* HDB and 59 publicly available reference genomes from diverse host niches. **(a)** Average Nucleotide Identity (ANI) heatmap generated using FastANI (v1.33) across 60 complete or high-quality *L. reuteri* genomes retrieved from NCBI RefSeq. Hierarchical clustering was performed based on pairwise ANI values to assess genomic relatedness. Species-level interpretation followed the standard ≥ 95-96% ANI threshold. **(b)** Pangenome composition generated using Roary (v3.13.0) with a 95% BLASTp identity cutoff. Genes were classified as core (present in ≥ 99% of strains), soft-core (95–99%), shell (15–95%), and cloud (< 15%). The distribution highlights a conserved core genome alongside a substantial accessory gene pool contributing to host-associated diversification. **(c)** Maximum-likelihood phylogeny constructed from the core genome alignment generated within the Roary pipeline. Tree inference was performed using IQ-TREE (v2.2.2) with 1,000 ultrafast bootstrap replicates. Bootstrap values are indicated at internal nodes. Host origin of each strain (human, rodent, poultry, porcine, food, environment) is color-coded. *L. reuteri* HDB clusters within the human-associated clade, consistent with ANI-based similarity.

To contextualize HDB strain among *L. reuteri* diversity, we compared it to 59 complete *L. reuteri* genomes from diverse hosts. Pairwise genomic relatedness by FastANI showed the highest identity to the *L. reuteri* DSM17938 reference 99.95%. The ANI distribution across the panel showed HDB firmly within the human-associated cluster, while more distant values were observed against rodent, porcine, and avian isolates, reflecting known host-linked stratification (Figure 5a).

Pangenome analysis (Roary, 95% BLASTp identity) across all 60 *L. reuteri* genomes identified a total of 11,725 gene families distributed across four categories. The core genome comprised 944 gene families (8.1% of pangenome) present in > 95% of strains, representing universally conserved functions including core metabolic pathways, DNA replication, transcription, and translation machinery essential for *L. reuteri* viability. The soft-core genome consisted of 171 gene families (1.5%) present in 95-99% of strains, likely reflecting near-universal accessory functions that are occasionally lost through genomic streamlining. The shell genome contained 1,912 gene families (16.3%) present in 5-95% of strains, representing variable accessory functions associated with host adaptation, carbohydrate utilization, and stress response that vary across lineages. The cloud genome was the largest partition, comprising 8,698 gene families (74.2%) present in fewer than 5% of strains, reflecting the extensive strain-level diversity characteristic of an open pangenome. The pangenome therefore remains open, consistent with ongoing gene flux driven by horizontal gene transfer and IS-mediated rearrangements. HDB contributed 9 singleton gene families (0.07% of pangenome), notably including a DMT family transporter, peptidoglycan recognition protein, response regulator transcription factor, and citrate lyase subunit alpha, suggesting strain-specific adaptations to the Indian infant gut environment (Figure 5b). Integration of functional annotation with pangenome structure revealed that core genes predominantly encode essential metabolic and genetic information processing functions, as reflected by the enrichment of COG categories L (replication), J (translation), and central carbohydrate metabolism pathways identified in KEGG. In contrast, shell and cloud genes contribute to accessory metabolic functions and hypothetical proteins, consistent with the large variable gene pool observed in Figure 5b. This functional stratification supports a conserved metabolic backbone with lineage-specific accessory diversification.

A maximum-likelihood phylogeny from core-gene alignments (IQ-TREE; 1,000 ultrafast bootstraps) placed HDB in a well-supported clade (100% bootstrap) with DSM 17938 and other human-derived strains, distinct from rodent/pig lineages. Branch lengths within the human clade were short (mean branch length ∼0.0036), indicating close relatedness at the core-genome level. The topology was concordant with the ANI heatmap, jointly supporting the inference that HDB is genomically closest to DSM 17938 among the surveyed references (Figure 5c).

Whole-genome synteny analysis further confirmed the close relationship between HDB and DSM 17938. MUMmer dot plot revealed near-perfect chromosomal collinearity across both ∼2.23 Mbp genomes, with a single main diagonal (slope ≈1) indicating conserved gene order and minimal structural rearrangements. Only minor local inversions (< 50 kbp) were visible as short anti-diagonals, with no evidence of large-scale inversions, translocations, or duplications (Supplementary Figure 3). This excellent synteny complements the high ANI (99.94%) and short core-genome branch lengths, collectively demonstrating that HDB represents intraspecies variation within *L. reuteri* rather than structural divergence indicative of a novel subspecies.

To further characterize the evolutionary history and genomic stability of *L. reuteri* HDB, prophage content and IS element distribution were systematically assessed. PHASTER analysis identified 9 prophage-related regions spanning approximately 137 kb in total, all classified as incomplete (*n* = 6, scores 30–60) or questionable (*n* = 3, score 70), with no intact prophage detected (Table 7). The largest region (Region 6; 42.1 kb; position 1,560,798-1,602,943) showed similarity to Corynephage Lederberg, while two questionable regions (Regions 8 and 9) matched Staphylococcal phages. The absence of intact prophage and the degraded state of all detected regions indicate that prophage remnants have undergone substantial mutational decay, consistent with long-term genomic stability.

**TABLE 7:** Prophage regions identified in the HDB genome.

Region	Region length	Completeness status	Score	#Total proteins	Region position	Most common phase	GC%
1	11.5 Kb	Incomplete	30	13	420809–432373	PHAGE Nodula vB NspS kac68v161 NC 048757(2)	37.52%
2	26.2 Kb	Incomplete	60	17	816298–842504	PHAGE_Bacill_vB_Ban$_Tsamsa_NC_023007(2)	38.02%
3	6.9 Kb	Incomplete	60	9	981737–988647	PHAGE_Escher_520873_NC_049344(2)	37.33%
4	5.9 Kb	Incomplete	60	7	1131345–1137295	PHAGE_Escher_520873_NC_049344(2)	37.25%
5	19.2 Kb	Incomplete	60	14	1522681–1541952	PHAGE_Bacill_G_NC_023719(3)	40.41%
6	42.1 Kb	Questionable	70	29	1560798–1602943	PHAGE_Coryne_Lederberg_NC_048790(6)	39.51%
7	10.2 Kb	Incomplete	60	13	1622468–1632680	PHAGE_Geobac_E2_NC_009552(4)	43.95%
8	7.6 Kb	Questionable	70	10	10 1798532–1806183	PHAGE_Staphy_phiPV83_NC_002486(2)	12.76%
9	7.4 Kb	Questionable	70	7	2102378–2109853	PHAGE_Staphy_StauST398_4_NC_023499(1)	39.13%

This table lists putative prophage regions predicted using PHASTER software. Table shows important parameters: Region completeness status, genomic coordinates, predicted protein counts, and closest known phage matches.

IS element analysis identified 178 IS elements across 9 families (Table 8), with IS30 representing the dominant family (*n* = 92, 51.7%), followed by ISL3 (*n* = 23, 12.9%), IS200/IS605 (*n* = 18, 10.1%), and ISLre2 (*n* = 12, 6.7%). Mapping IS element positions against MUMmer alignment breakpoints relative to DSM17938 revealed that 14 of 23 breakpoints (61%) exhibited IS element enrichment above the genome-wide background density (8.0 elements per 50 kb window), with the highest enrichment observed at breakpoints flanking the major ∼200 kb inversion region (900-1,200 kb; Supplementary Figure 4). The predominance of IS30-family elements and their spatial association with structural breakpoints suggests IS-mediated recombination as a plausible mechanism for the intraspecific structural variation observed between HDB and DSM17938.

**TABLE 8:** Insertion sequence (IS) family distribution in the *L. reuteri* HDB genome.

IS family/mobile element	Count
1S30	92
ISL3	23
1S200/1S605	18
ISLre2	12
IS110	9
IS3	7
1S21	6
IS66	5
1S1182	5
Total	178

This table shows list of Insertion sequence (IS) families identified using ISfinder. Counts represent total copies detected across the HDB genome.

### Evolutionary selection analysis of core genome across host niches

**FIGURE 6:**
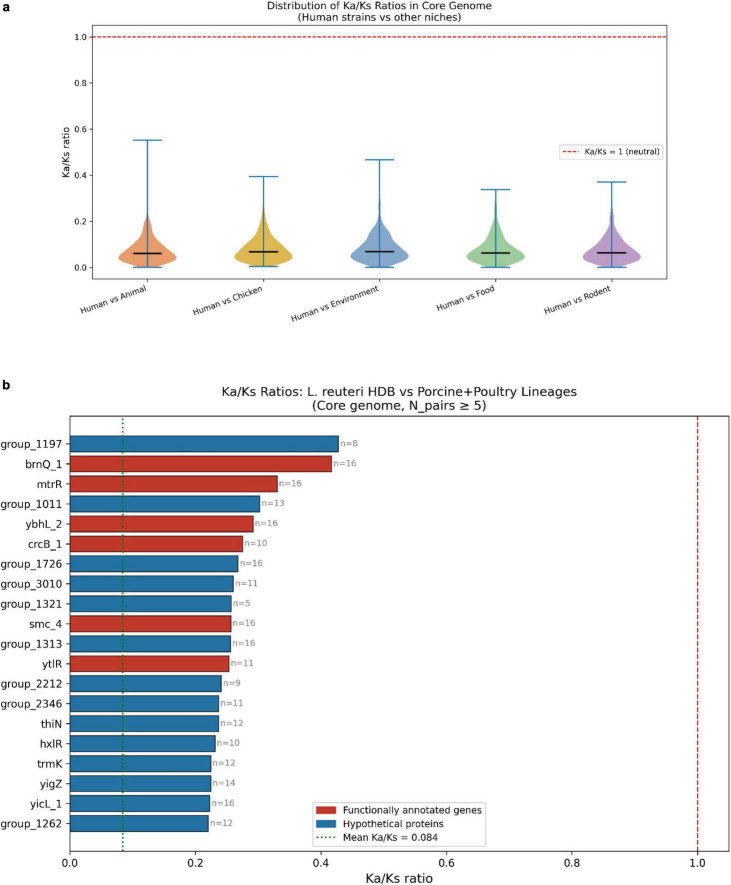
Evolutionary constraint analysis of core genes in *Limosilactobacillus reuteri*. **(a)** Distribution of dN/dS (Ka/Ks) ratios for orthologous core genes comparing human-associated strains with animal (porcine), poultry, environmental, food, and rodent lineages. Core genes were identified using Roary (95% BLASTp cutoff; ≥ 99% strain presence). Ka/Ks ratios were estimated from codon-based alignments after excluding zero dS values. The dashed red line indicates Ka/Ks = 1 (neutral evolution), and black bars denote medians. Most core genes exhibit Ka/Ks < 1, consistent with purifying selection. **(b)** Gene-level Ka/Ks values for *L. reuteri* HDB compared with porcine and poultry lineages (core genes with ≥ 5 pairwise comparisons). Bars represent mean Ka/Ks per ortholog group; the green dashed line indicates the overall mean (0.084), and the red dashed line marks neutrality (Ka/Ks = 1). Annotated genes and hypothetical proteins are distinguished by color. No core genes show evidence of strong positive selection.

To investigate adaptive evolution specific to the human infant gut niche, Ka/Ks (dN/dS) ratios were calculated across 944 core genes comparing human-associated *L. reuteri* strains against host-specialized porcine (*n* = 10) and poultry (*n* = 7) lineages using the Yang-Nielsen (YN00) method. Across all pairwise comparisons, core gene Ka/Ks ratios were uniformly well below 1.0 (mean = 0.084; median = 0.071), indicating that the *L. reuteri* core genome is subject to strong purifying selection regardless of host origin, consistent with the functional indispensability of conserved core genes (Figure 6a). The broader human-associated clade showed a mean Ka/Ks of 0.079 across niche comparisons, while *L. reuteri* HDB exhibited a slightly elevated mean Ka/Ks of 0.084 relative to porcine and poultry lineages, suggesting proportionally more non-synonymous substitutions have accumulated in HDB compared to animal-associated strains (Supplementary Figure 5).

Gene-level analysis of HDB specifically against porcine and poultry lineages identified several functionally annotated genes with the highest relative Ka/Ks values (Figure 6b), including brnQ (branched-chain amino acid transporter), mtrR (transcriptional repressor of efflux pumps), smc (chromosome segregation), crcB (fluoride/ion channel), and ytlR (sugar metabolism regulator). Although all values remained below the positive selection threshold (Ka/Ks < 1), the relatively elevated non-synonymous substitution rates in these transport, stress-response, and regulatory genes suggest localized relaxation of purifying selection consistent with gradual functional specialization to the nutritional and physiological pressures of the human infant gut niche. A complementary analysis of human-associated strains broadly versus environmental isolates identified apbE (thiamine biosynthesis), yabA (DNA replication), gspA (general stress protein), and kefG (potassium efflux) among the highest-ranked core genes (Supplementary Figure 6), further supporting the inference that stress tolerance, ion homeostasis, and cofactor metabolism loci are evolving at comparatively faster rates in human-adapted lineages.

### Metabolic pathway completeness and functional potential

KEGG mapping revealed ∼70% pathway coverage across major metabolic subsystems, with near-complete pathways for glycolysis (66.7%), pentose phosphate pathway (70.0%), and pyruvate fermentation (75.0%). Amino-acid biosynthesis was complete for lysine and alanine/aspartate/glutamate, and partial for aromatic amino acids, consistent with nutrient-rich gut environments and cross-feeding. Carbohydrate utilization modules encompassed PTS and ABC transporters for maltose, fructose, raffinose/stachyose, and lactose, reflecting broad saccharolytic capacity. Presence of several lactate dehydrogenases and acetate kinase genes confirmed strong fermentative metabolism typical of *Limosilactobacillus* (Table 9).

**TABLE 9:** Major metabolic pathways and their genes.

Metabolic pathways	Enzymes/genes involved
Glycolysis/Embden-Meyerhof-Parnas	*Pfk, foa, gapA, tpiA, pgk, gpmA, eno, pyk*
Pentose phosphate pathway	*zwf, pgl, gnd, rpiA, rpe, tkt, tal*
Pyruvate fermentation	*pflA, pflB, adhE, Idh, ackA*
Lysine biosynthesis	*dapA, dapB, dapD, dapC, dapE, IysA*
Alanine/aspartate/glutamate metabolism	*aspC, asnB, gltB, gltD, gudB*
Aromatic amino acids (partial)	*aroA, aroB, aroC, aroE*
Maltose utilization	*malP, MalQ, malL*
Fructose utilization	*fruA, fruK*
Raffinose/stachyose utilization	*rafA, rafB, nsmE*
Lactose utilization	*lacZ, lacY*

This table contains list of pathways involved in *L. reuteri* HDB metabolism with their respective genes.

The strain displayed enriched Vitamin and cofactor synthesis pathways, particularly large clusters of cob/cbi genes (245 cob/cbi-related KOs) for cobalamin (B_12_) production, folate pathway enzymes, and a complete heme biosynthesis gene set. Due to the pdu operon, having all 6 genes (pduCDEQP), the strain is capable of conversion of glycerol to 3-hydroxypropionaldehyde (reuterin), a key antibacterial metabolite linked with antagonism against enteric pathogens. Together, these modules underscore metabolic versatility that can enhance colonization, niche competition, and microbe-host interactions in the infant gut (Table 10).

**TABLE 10:** Vitamins, Cofactors and secondary metabolites synthesis pathways and their genes.

Metabolic pathways	Enzymes/genes involved
Cobalamin (B_12_) biosynthesis	*cobB, cobC, cobD, cobE, cobF, cobG*
Folate biosynthesis	*folP. folC, folA*
Heme biosynthesis	*hemA, hem, hemB, hemC, hemD, hemE, hemF*
Propanediol utilization (pdu operon)	*pduC, pduD, pduE, pduQ, pduP. pduR*

This table contains genes involved in Cobalamin (vitamin B_12_), folate, heme biosynthesis, and propanediol utilization (pdu operon) pathways based on KEGG and locus synteny analysis.

Subsystem classification by RAST assigned genes to 28 functional categories, covering 30% of the genome. The most represented categories were Protein Metabolism (132 genes), Amino Acids and Derivatives (112), Carbohydrates (111), and Cofactors/Vitamins/Prosthetic Groups (89) (Supplementary Figure 7). BV-BRC annotations corroborated these findings and additionally flagged loci associated with oxidative stress mitigation (e.g., thioredoxin/peroxidase), metal ion homeostasis (e.g., ABC-type transporters, metalloregulators), and cell-surface architecture (e.g., LPXTG-anchored proteins), which may contribute to mucosal adherence and immune crosstalk (Supplementary Figure 8). These pathway enrichment findings were further interrogated at the systems level through genome-scale metabolic modeling, as described in the following section.

### Genome-scale metabolic modeling and flux balance analysis

To functionally contextualize the enriched carbohydrate and cofactor metabolic pathways identified in *L. reuteri* HDB, genome-scale metabolic models (GEMs) were reconstructed for HDB and two global reference strains (JCM1112 and DSM17938) using ModelSEED via the KBase platform. Flux Balance Analysis (FBA) predicted growth rates (objective values) of 12.40, 13.94, and 12.40 mmol/gDW/h for HDB, JCM1112, and DSM17938, respectively, under identical complete media conditions, indicating broadly comparable metabolic capacity across strains (Supplementary Figure 9).

**FIGURE 7:**
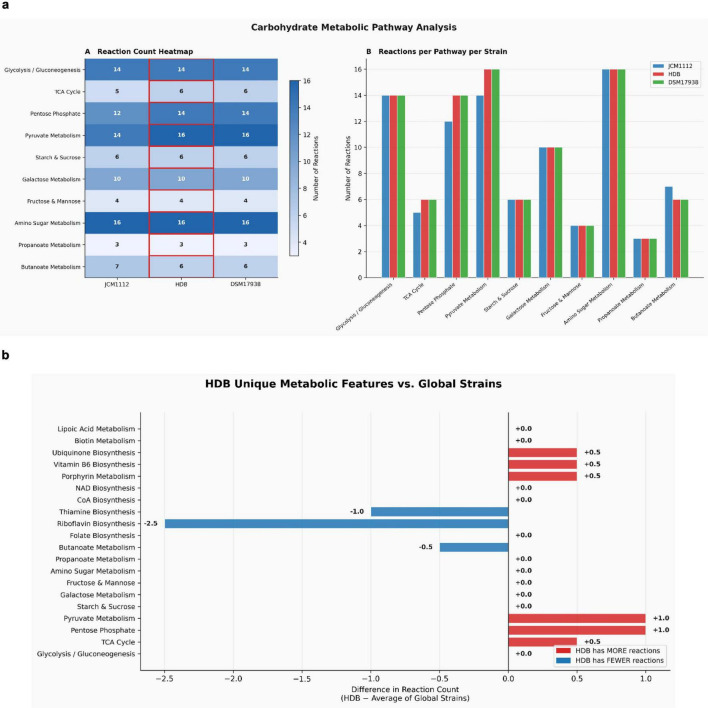
Comparative genome-scale metabolic reconstruction of *Limosilactobacillus reuteri* HDB and reference strains. **(a)** The figure shows comparison of reaction counts across major carbohydrate metabolic pathways in *L. reuteri* HDB, JCM1112, and DSM 17938. Genome-scale metabolic models were reconstructed using ModelSEED in the KBase platform after RASTtk annotation. Reactions were mapped to KEGG pathways, and the total number of reactions was calculated for carbohydrate pathways. **(b)** The figure explains difference in reaction counts of HDB compared to the average of the reference strains. Positive values indicate relatively higher reaction numbers in HDB, while negative values indicate fewer reactions. Slight enrichment is observed in the pentose phosphate and pyruvate pathways, whereas lower representation is seen in riboflavin and some cofactor biosynthesis pathways. These values represent predicted metabolic capacity based on *in silico* analysis.

Comparative analysis of pathway-level reaction content revealed that HDB harbors an expanded carbohydrate metabolic network relative to global strains, with additional reactions annotated to the Pentose Phosphate Pathway, TCA Cycle, and Pyruvate Metabolism (Figure 7a). These findings are consistent with the near-complete KEGG pathway coverage observed in glycolysis (66.7%), pentose phosphate (70.0%), and pyruvate fermentation (75.0%) reported above, and provide functional support for the genomically inferred saccharolytic capacity of HDB.

In the cofactor biosynthesis domain, HDB showed increased predicted metabolic flux capacity in Porphyrin biosynthesis, Vitamin B6 biosynthesis, and Ubiquinone biosynthesis pathways compared to global reference strains (Figure 7b). This is consistent with the large cobalamin biosynthesis gene cluster and the complete heme biosynthesis module identified in HDB. Conversely, HDB showed relatively fewer reactions in Riboflavin and Thiamine biosynthesis pathways compared to JCM1112, suggesting some cofactor pathway specialization between strains (Supplementary Figure 10). Taken together, these GEM-based predictions provide a systems-level metabolic perspective supporting the hypothesis that *L. reuteri* HDB possesses enhanced functional potential in carbohydrate utilization and cofactor metabolism key metabolic traits relevant to its adaptation and performance in the infant gut environment.

### Host-specific adaptation signals and strain-specific features

Given the established host-linked population structure of *Limosilactobacillus reuteri*, we examined strain HDB for genomic signatures indicative of human infant gut adaptation. The surface protein repertoire revealed a suite of adhesion and colonization factors, including *gap* and *eno* genes, adhesion determinants (mapA, ef-tu), LPXTG-motif sortase substrates, and exopolysaccharide (EPS) biosynthesis genes. These features are functionally associated with mucosal adherence, epithelial barrier modulation, biofilm formation, and immunomodulatory interactions within the host gastrointestinal tract.

Apart from these adhesion qualities, HDB harbors classical stress-response proteins (chaperons) groEL, dnaK, and clpC along with bile salt hydrolase (bsh). These give it tolerance against bile, acid and osmotic fluctuations that Indian infants face during early gut settling and milk-to-solid food shift.

Pangenome analysis further revealed several gene families unique to HDB, including DMT transporters, response-regulator transcription factors, and proteins related to peptidoglycan. These features may indicate subtle ecological adaptation to the infant gut and the dietary inputs typical of early life. Although these predictions need experimental confirmation, the combination of probiotic-associated adhesion and stress-tolerance genes, distinct genomic markers, and its placement within the human-associated *L. reuteri* lineage suggests that HDB functions as a human-associated lineage ecotype with traits that support colonization and interaction in the infant intestine.

To determine whether the sucrase-type levan pathway identified in HDB represents a conserved feature of the species or a strain-specific adaptation, a comparative presence-absence analysis of EPS and levan biosynthesis loci was performed across all 60 *L. reuteri* genomes grouped by host niche (Supplementary Figure 11). The core levan biosynthesis module comprising levansucrase (levS), glycosyltransferases (gtfA_1, gtfA_2), UDP-glucose 4-epimerase (galE), and dTDP-glucose 4,6-dehydratase (rmlB_1) was conserved in the majority of human-associated strains (> 80%, *n* = 24 of 30), including the reference probiotic strain DSM17938, indicating that this pathway is a characteristic feature of human-adapted *L. reuteri* rather than a unique acquisition in HDB. Structural cell wall genes rodA, ftsW, and murJ showed near-universal conservation across all host groups, consistent with their essential roles in peptidoglycan biosynthesis. In contrast, levS and rmlB_1 showed the most variable distribution across non-human lineages, being reduced or absent in several rodent, porcine, and environmental isolates. This host-stratified distribution indicates that the sucrase-type levan pathway is a lineage-associated accessory feature enriched in human-adapted strains. It is therefore likely a conserved trait within the human clade, rather than a strain-specific acquisition unique to HDB.

## Discussion

The phylogenomic assessment brings out a few features of HDB that are central to understanding its position within the *L. reuteri* group. The strain falls within the human-associated clade (dDDH 69.7% to subspecies porcinus) and stands apart from several food-industry strains, pointing to a different evolutionary trajectory. Its genome also maintains the complete pdu and cob-hem operons, consistent with the ability to produce both reuterin and B_12_ which traits commonly linked to adaptation in the infant gut. On the safety side, we did not detect virulence factors, hemolysis-related genes, or mobile antibiotic-resistance elements. While these findings will need to be backed up through adhesion studies and animal experiments, the overall picture suggests that HDB carries the genomic qualities expected of a strain suited for early-life microbiota support.

The broader genome-based comparisons, including ANI, core-genome phylogeny and TYGS dDDH, place HDB comfortably within the human-associated cluster of *L. reuteri*, with its closest relationship to strains already used in clinical contexts. The agreement across these different analyses strengthens confidence in the taxonomic placement yet also draws attention to the fluid boundaries within the *L. reuteri* species complex. TYGS-based dDDH values that hover around classical species and subspecies cut-offs illustrate how difficult fixed thresholds become when sampling across hosts is uneven. Rather than a flaw in the method, this reflects the biology of the organism: *L. reuteri* lineages show historical host-linked structure but also occasional cross-host movement or retention of shared ancestral variation. The short branch lengths within the human clade point to relatively recent divergence, and the core genome remains largely conserved across strains, while the accessory genome especially metabolic loci seem to drive ecological differences. These patterns reinforce the idea that taxonomic assignment in this group is better viewed as part of a genomic continuum rather than strict categorical divisions.

Complementing this phylogenomic placement, core-genome selection analysis further supports evolutionary stability within the human-associated lineage. Across 944 conserved genes, mean Ka/Ks ratios remained uniformly below 0.1 across all host niche comparisons, indicating strong purifying selection and preservation of essential cellular functions. Even the highest-ranking transport and regulatory loci in HDB (e.g., *brnQ*, *mtrR*) exhibited values well below neutrality, suggesting incremental divergence rather than adaptive shifts. Together with near-collinear whole-genome alignment and IS30-enriched but localized inversions, these results indicate that HDB diversification reflects insertion sequence-mediated recombination and gradual lineage-level drift rather than large-scale structural or functional innovation.

The KEGG and COG annotations indicate that HDB is reasonably equipped to handle the shifting carbohydrate environment typical of early life. The presence of transporters and enzymes for milk oligosaccharides as well as for more common dietary sugars suggests that the strain can adapt quickly as substrate availability changes during weaning, a pattern also noted in earlier work (Spinler et al., 2014). Alongside this, the genome shows partial pathways for aromatic amino acids and full pathways for a few essential amino acids, pointing to a mixed metabolic strategy in which the strain synthesizes some compounds on its own while depending on dietary inputs or neighboring microbes for others. Such metabolic give-and-take aligns well with the current understanding of infant gut ecology, where interdependence among microbes supports efficient colonization and early community development (Magnúsdóttir et al., 2017; Milani et al., 2017; Pacheco et al., 2019; Stewart et al., 2018).

Genome-scale metabolic reconstruction and pathway-level comparison indicate that HDB retains metabolic architecture highly similar to reference human-associated strains. Predicted growth rates under standardized *in silico* conditions were comparable to JCM1112 and DSM 17938, while reaction-level analysis revealed modest quantitative differences in carbohydrate and porphyrin-associated pathways rather than expanded metabolic scope. KEGG mapping showed near-complete glycolytic and pentose phosphate modules, consistent with conserved saccharolytic capacity. The intact 38.5 kb *pdu-cob-hem* region integrates propanediol utilization with cobalamin and heme biosynthesis, reflecting preserved lineage-level metabolic organization rather than strain-specific innovation (Santos et al., 2008; Sriramulu et al., 2008; Talarico et al., 1988). This pattern also helps explain why human-associated *L. reuteri* commonly retain intact *pdu* and *cob* pathways, whereas these regions are frequently reduced or missing in strains adapted to non-human hosts (Spinler et al., 2008; Wegmann et al., 2015).

The combination of antimicrobial activity and vitamin-linked metabolism in HDB makes the strain particularly interesting from both ecological and host-health perspectives. The genomic evidence for reuterin (broad-spectrum antimicrobial) formation indicates that the strain could, under suitable conditions, limit the growth of undesirable organisms in the infant gut (Santos et al., 2008; Spinler et al., 2008; Talarico et al., 1988). In practice, though, such activity will depend very much on how much glycerol is available, the local physiology, and the surrounding microbiota, since reuterin is neither selective nor uniformly produced. The capacity for cobalamin synthesis is also notable, as very few gut commensals carry a complete pathway; this feature could influence B_12_ balance in the gut or shape interactions with microbes that rely on it as a cofactor (Santos et al., 2008; Sriramulu et al., 2008). These observations, however, remain predictive. It will be necessary to demonstrate actual production of reuterin and B_12_ in conditions that reflect the infant gut and to determine whether any of these metabolites reach the host in a usable form (Kullen and Klaenhammer, 1999; Marco et al., 2006; Sanders et al., 2018).

The genomic landscape of HDB shows a coherent set of traits that are usually associated with persistence in the infant gut. The strain carries multiple adhesion systems like moonlighting proteins, LPXTG-anchored substrates and EPS-related genes which together point to a robust mucosal association capacity (Bron et al., 2012; Buck et al., 2005; Deepika et al., 2009; Lee et al., 2013). In addition, the presence of bile salt hydrolase and several stress-response chaperones indicates a reasonable ability to withstand gastric acidity and bile exposure during transit (Bron et al., 2012; Lee et al., 2013). Taken together, these signatures present a credible case for ecological fitness; however, as always, these predictions will need to be strengthened through direct assays of adhesion, acid-bile tolerance and competitive behavior against representative infant-gut microbes (Marco et al., 2006; Sanders et al., 2018).

Safety becomes even more critical when a probiotic is intended for infants, and the genomic data for HDB is reassuring in this regard. We did not detect any transferable antibiotic-resistance genes (ARGs), plasmids, or recognized virulence factors in its genome, which together support the view that the strain carries a relatively low safety risk (Sanders et al., 2019). The Probiotic Potential Risk Score (PPRS) of 1 further supports this assessment. Additionally, the genome does not carry any CRISPR-Cas systems, and the absence of CRISPR-Cas systems suggests reliance on alternative phage-defense mechanisms; however, regulatory safety assessment remains dependent on phenotypic validation. While such systems can help a bacterium deal with phage attacks, their absence means there is far less concern about horizontal gene transfer or unwanted genomic rearrangements when the strain is used in production settings (Fontana et al., 2013; Hill et al., 2014; Sanders et al., 2019). Still, genomic safety assessment is necessary but not sufficient: phenotypic antibiotic susceptibility testing, *in vitro* cytotoxicity assays, and controlled animal studies will be required before any clinical use is contemplated (Sanders et al., 2018; Sanders et al., 2019).

Although HDB contributes few singleton families to the species pangenome, those unique genes (e.g., DMT transporters, peptidoglycan recognition proteins, citrate lyase components) may encode fine-scale ecological specializations (Duar et al., 2017; Frese et al., 2013; Gänzle, 2015; Qin et al., 2014; Tettelin et al., 2008). Such low-frequency variation is typical in bacteria occupying tightly constrained host niches, where horizontal gene flux and local selection can generate adaptive innovations (Qin et al., 2014; Tettelin et al., 2008). Mapping the geographic and temporal distribution of these alleles through expanded sampling of Indian infant microbiomes would clarify whether these features represent local adaptation, transient acquisitions, or neutral variation. Population-level sampling would also resolve how representative HDB is of Indian infant *L. reuteri* and identify lineage-specific genomic traits that correlate with health or environmental variables (Duar et al., 2017; Frese et al., 2013; Qin et al., 2014).

The successful generation of a complete, closed genome using hybrid sequencing validates the approach for complex lactic acid bacterial genomes containing repetitive surface proteins, insertion sequences, and rRNA operons (De Maio et al., 2019; Watson and Warr, 2019; Wick and Holt, 2022; Wick et al., 2017). The integration of high-accuracy short reads with long-read scaffolding overcame the limitations of individual sequencing platforms, producing an assembly suitable for comprehensive comparative genomics and accurate identification of mobile genetic elements. Future studies may benefit from enhanced plasmid detection methods, including targeted plasmid enrichment or long-range optical mapping, to ensure complete characterization of extrachromosomal elements that might be present at low copy numbers or under specific growth conditions (Wick and Holt, 2022).

This study is constrained primarily by its single-isolate design and reliance on genomic inference for functional claims. Priorities for future work include: (a) experimental verification of reuterin and B_12_ production under gut-relevant conditions (quantified by LC-MS/GC-MS and isotope labeling where possible); (b) *in vitro* and *in vivo* assays to demonstrate adhesion, colonization, immunomodulation, and pathogen antagonism; (c) antibiotic susceptibility profiling and safety assessments in relevant preclinical models; and (d) broader population genomics of Indian infant *L. reuteri* to contextualize HDB within regional diversity (Kullen and Klaenhammer, 1999; Lee and Tannock, 2021; Leulier et al., 2017; Marco et al., 2006; Sanders et al., 2018). In addition, to understand how the strain behaves in conditions that mimic the gut, we will examine its metabolome and transcriptome during growth on physiologically relevant substrates. This should help reveal when key genes are switched on and how metabolic flux moves through the *pdu-cob-hem* axis.

In this work, we combined high-quality genome assembly with comparative analysis to position HDB within the human-associated *L. reuteri* group. The analysis highlights key features supporting its survival in the infant gut, such as a complete reuterin pathway, extensive B_12_ biosynthesis genes, and multiple adhesion modules. Along with a favorable genomic safety profile, these features are consistent with those reported in established human-associated *L. reuteri* strains. However, functional studies are required to confirm their probiotic performance (Sanders et al., 2016; Walter et al., 2018; Zheng et al., 2015). Although strain HDB shows genomic features commonly associated with probiotic *L. reuteri* strains, comparative analysis revealed high overall conservation with the reference strain DSM 17938. This suggests that HDB represents a geographically relevant reference isolate within a conserved human-associated lineage rather than a functionally divergent strain. However, these genomic predictions must be validated through laboratory testing and efficacy studies, keeping in mind infant digestion and feeding practices. Overall, this study demonstrates how genome-level insights can improve our understanding of host-microbe adaptation and guide the selection of strains suited for Indian populations, where such data is often limited.

## Data Availability

The whole (hybrid-assembly) genome of *Limosilactobacillus reuteri* strain HDB has submitted in the Indian Nucleotide Data Archive (INDA) under the study accession INRP000518 and in the INSDC/European Nucleotide Archive (ENA) under BioProject PRJEB105324 (study accession ERP186527). The corresponding genome assembly is publicly available under the accession GCA_977963825.1. The Analyses presented in this study are based on the assembled genome sequence. A total of 59 publicly available *Limosilactobacillus reuteri* whole genomes (Assembly status: Complete) representing diverse host origins were included for comparative analyses. Strain names, accession numbers, and isolation sources are provided in Supplementary Table 1.

## Footnotes

1
https://pubmlst.org/

2
https://mummer.sourceforge.net/

3
https://phaster.ca

4
https://isfinder.biotoul.fr
