# Supplementary material for: Genome characterization and comparative genomics of Limosilactobacillus reuteri HDB isolated from the gut of an Indian infant
Source: Front Microbiol. 2026 Apr 17;17:1780782. doi: 10.3389/fmicb.2026.1780782 (PMC13132842; doi:10.3389/fmicb.2026.1780782)
Supplement: Supplementary file 1 [file Data_Sheet_1.DOCX]

**Supplementary figures:**

**Supplementary Fig. S1. 16S rRNA BLAST similarity results**

The figure depicts BLASTn results for the 1,45 bp 16S rRNA gene sequence of *Limosilactobacillus reuteri* HDB against the NCBI RefSeq database. The top hits show 99.56% sequence identity with 100% query coverage, supporting species-level identification.

**Supplementary Fig. S2. Ribosomal multilocus sequence typing (rMLST) species assignment output**

The given analysis was performed using 53 ribosomal protein genes through the PubMLST database. The isolate was assigned to *Limosilactobacillus reuteri* with 100% support.

**Supplementary Fig. S3. Whole-genome alignment of *L. reuteri* HDB and DSM 17938**

Dot-plot alignment generated using MUMmer showing large-scale synteny between the complete genomes of HDB and DSM 17938. The continuous diagonal pattern indicates conserved gene order and high chromosomal collinearity between the two strains.

**Supplementary Fig. S4. IS element distribution and structural rearrangement analysis**

Insertion sequences were identified using ISfinder. Panel A shows the genomic positions of different IS families across the HDB chromosome. Panel B displays IS density calculated in 50 kb windows along the genome. Panel C shows the enrichment of IS elements at whole-genome alignment breakpoints in comparison with DSM 17938.

**Supplementary Fig. S5. Mean Ka/Ks ratios per niche comparison**

This figure presents the mean dN/dS (Ka/Ks) values for core genes across different host niche comparisons. Values are shown as mean ± SEM. In all cases, Ka/Ks values remain below 1, indicating predominant purifying selection across niches.

**Supplementary Fig. S6. Top 20 core genes ranked by dN/dS (Ka/Ks) (Human vs Environment)**

This figure shows the gene-level Ka/Ks values for the top 20 core genes with the highest ratios in the comparison between human-associated and environmental strains. The dashed red line indicates the neutrality threshold (Ka/Ks = 1). All genes remain under purifying selection.

**Supplementary Fig. S7. RAST subsystem annotation overview**

Functional subsystem classification generated using the RAST annotation server, summarizing distribution of genes across metabolic and cellular categories.

**Supplementary Fig. S8. COG functional category distribution (BV-BRC annotation)**

Cluster of Orthologous Groups (COG) classification derived from BV-BRC annotation, showing proportional representation of functional categories within the HDB genome.

**Supplementary Fig. S9. Flux Balance Analysis (FBA) predicted growth rates and total reactions**

Genome-scale metabolic models reconstructed in KBase (ModelSEED framework) were subjected to Flux Balance Analysis under complete-medium conditions. Predicted growth rate (objective value) and total reaction counts are shown for HDB, JCM1112, and DSM 17938.

**Supplementary Fig. S10. Cofactor metabolic pathway reaction counts**

Comparison of reaction counts across cofactor biosynthesis pathways based on KEGG mapping from reconstructed genome-scale metabolic models.

**Supplementary Fig. S11. Distribution of EPS and levan biosynthesis genes across *L. reuteri* strains**

Presence/absence heatmap of EPS-related loci, including levansucrase (levS), across 60 L. reuteri genomes grouped by host niche. Gene presence was determined from annotated ortholog groups.

**Supplementary Table:**

**Supplementary Table 1.** List of publicly available whole genome dataset used for comparative analysis

**Supplementary figures:**

**
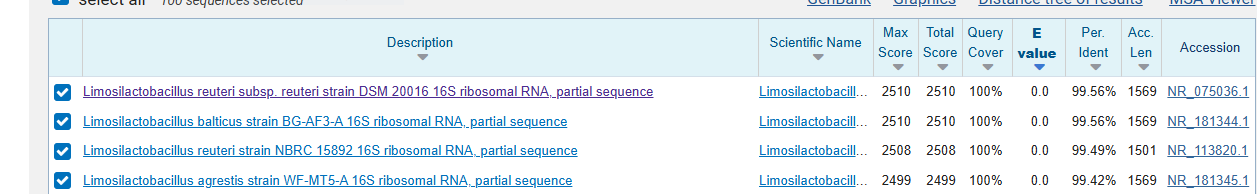
**

(Supplementary Fig. S1)

**
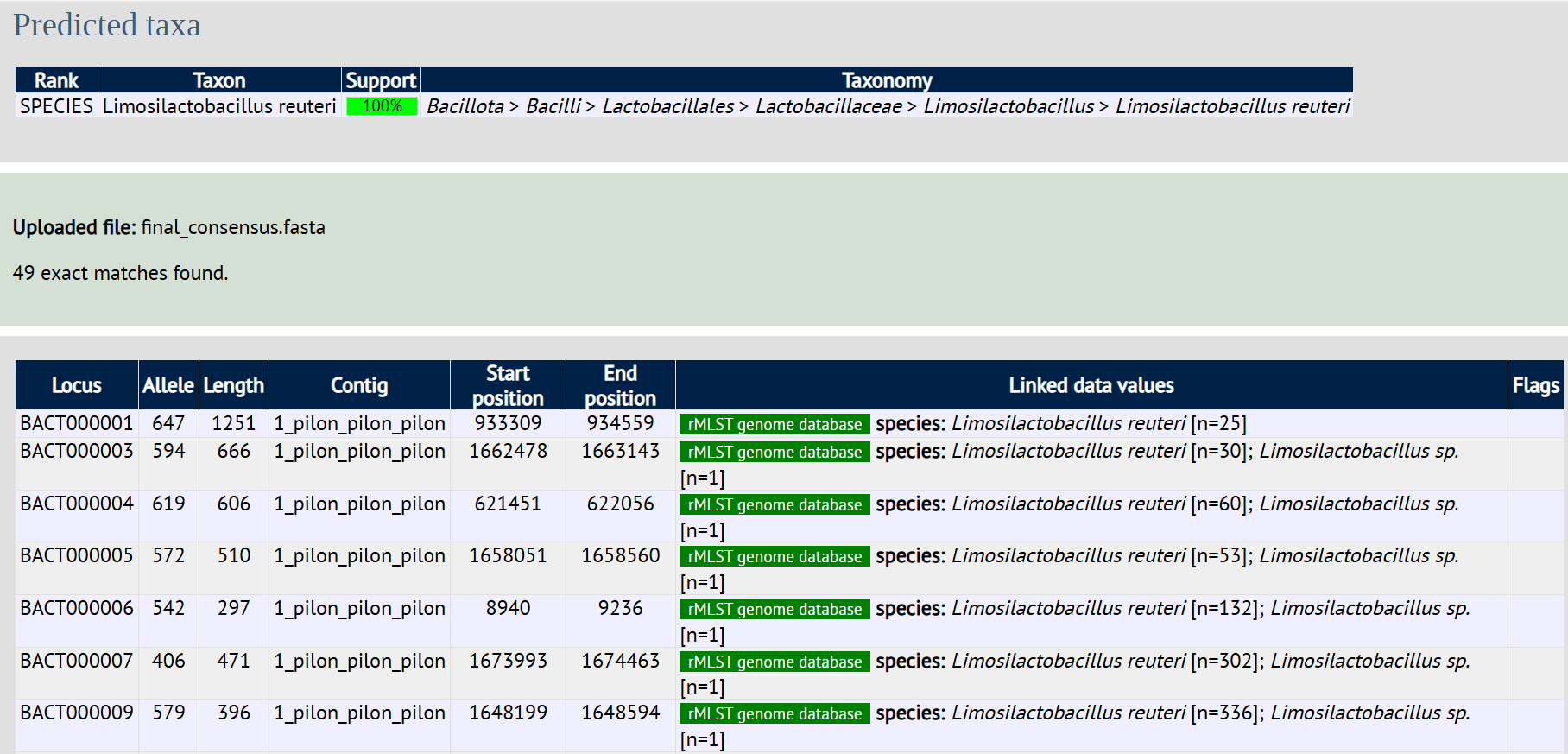
**

(Supplementary Fig. S2)

**
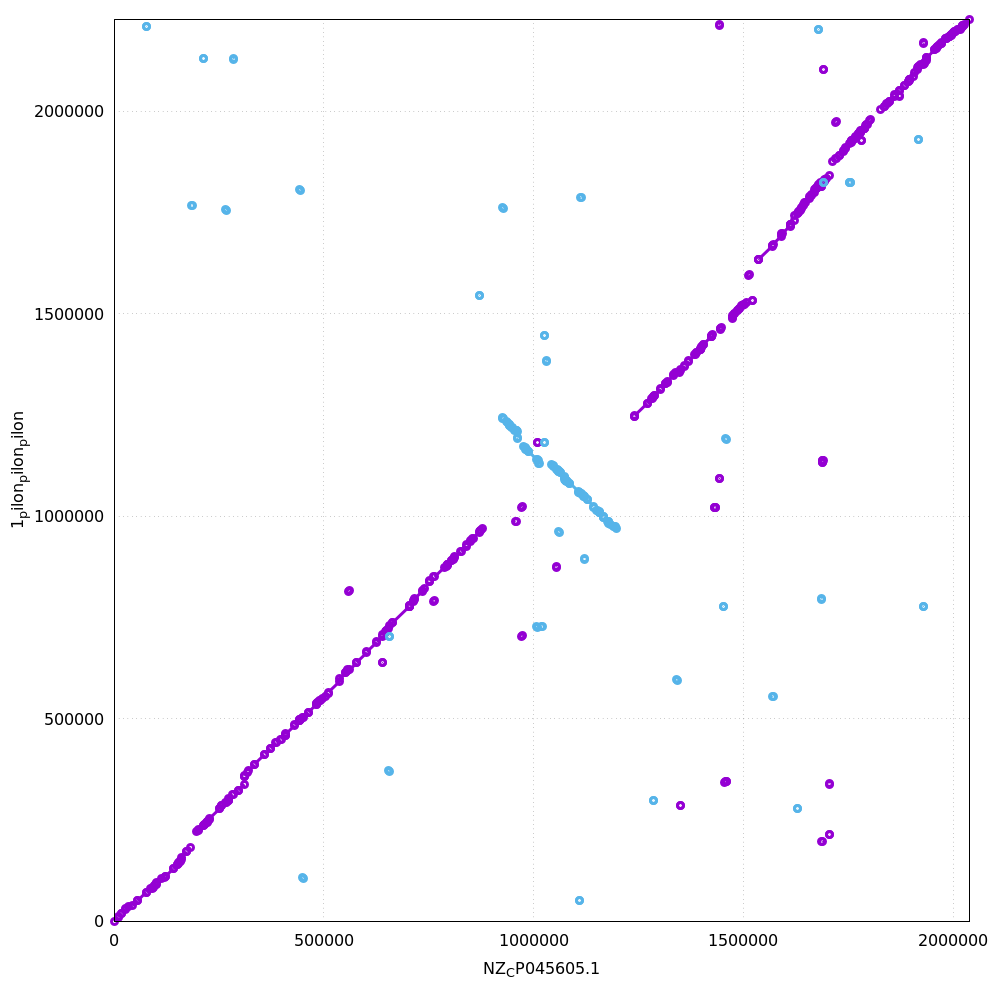
**

(Supplementary Fig. S3)


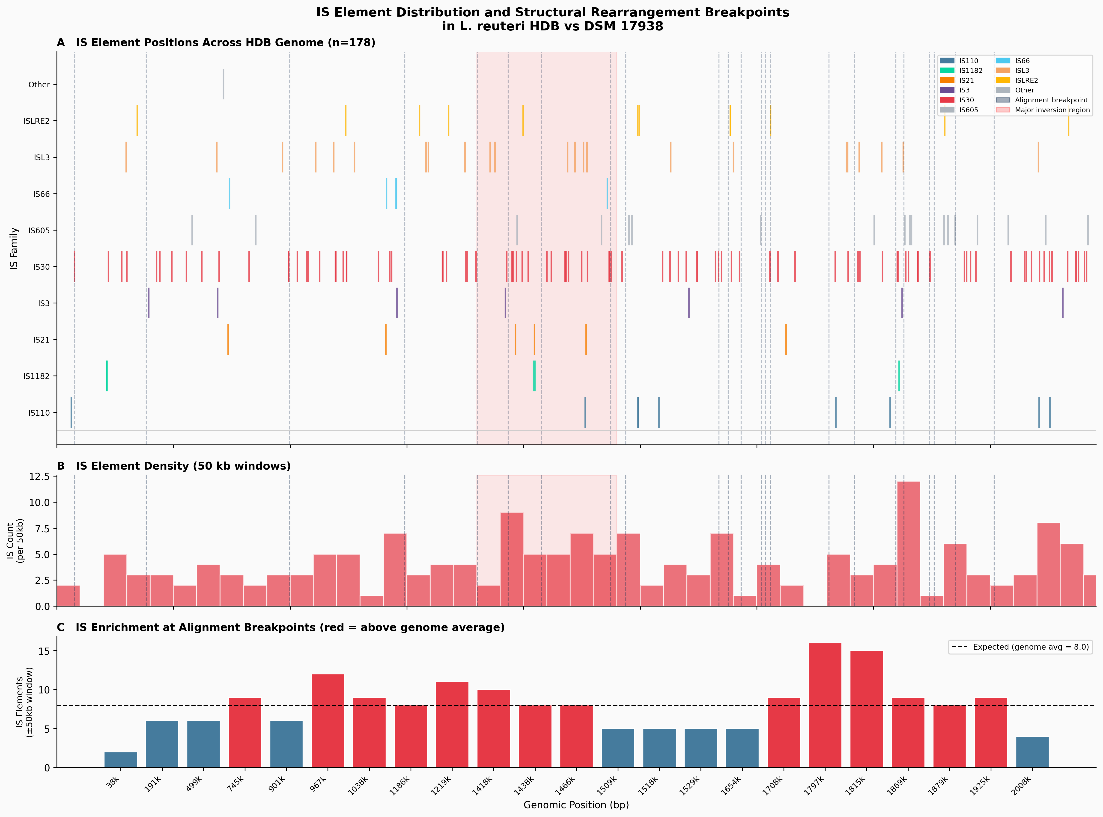


(Supplementary Fig. S4)


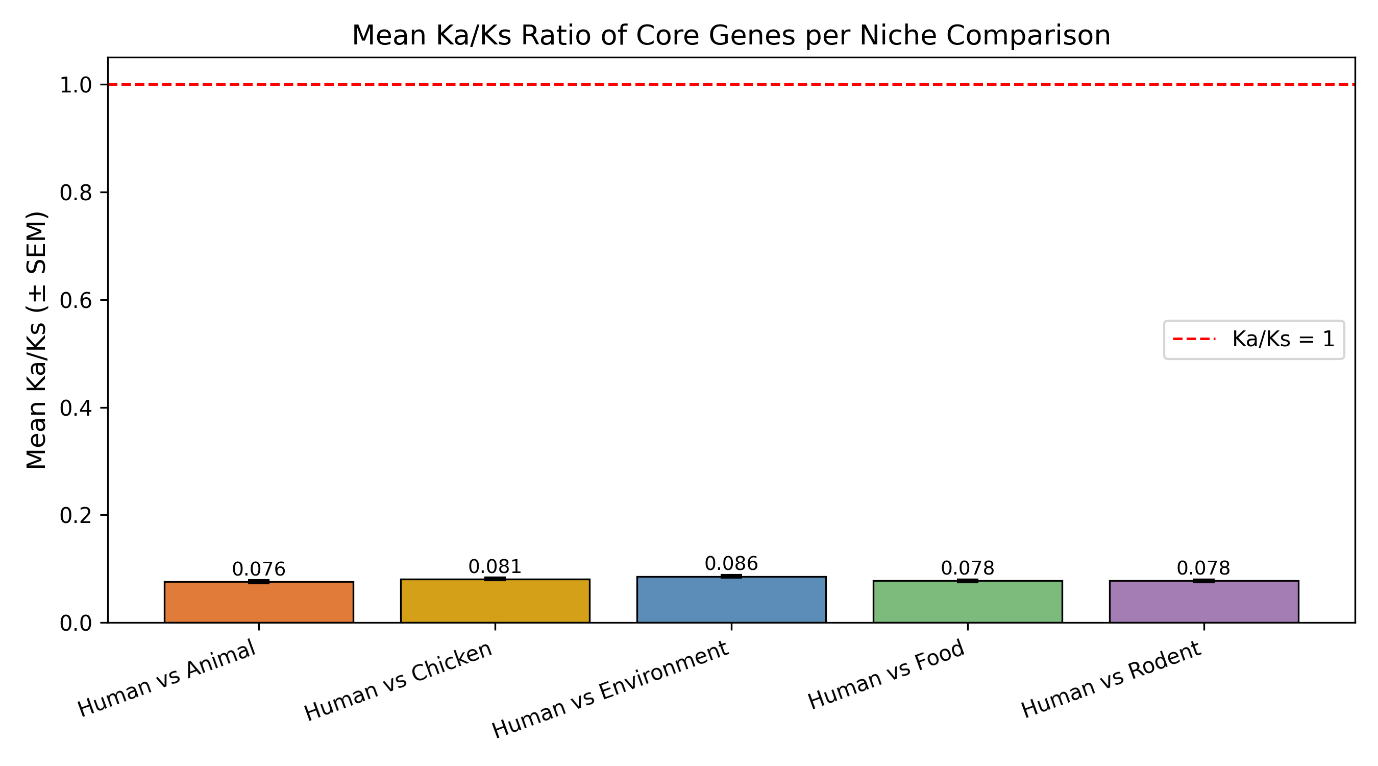


(Supplementary Fig. S5)


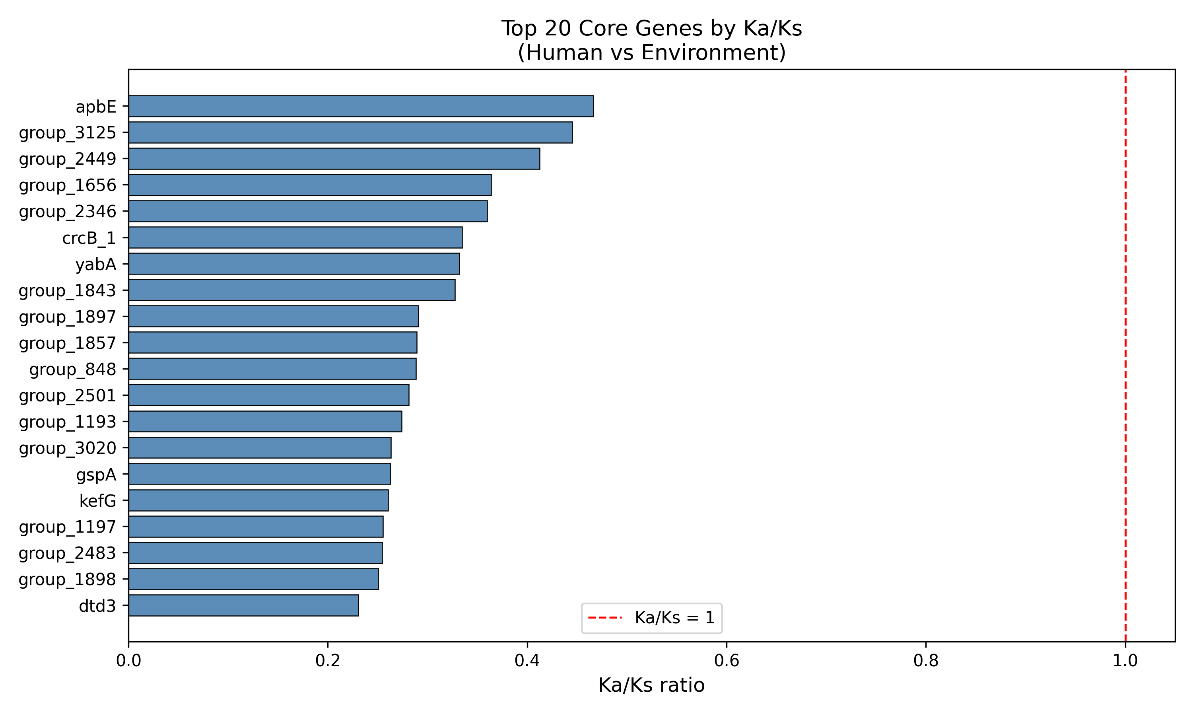


(Supplementary Fig. S6)

**
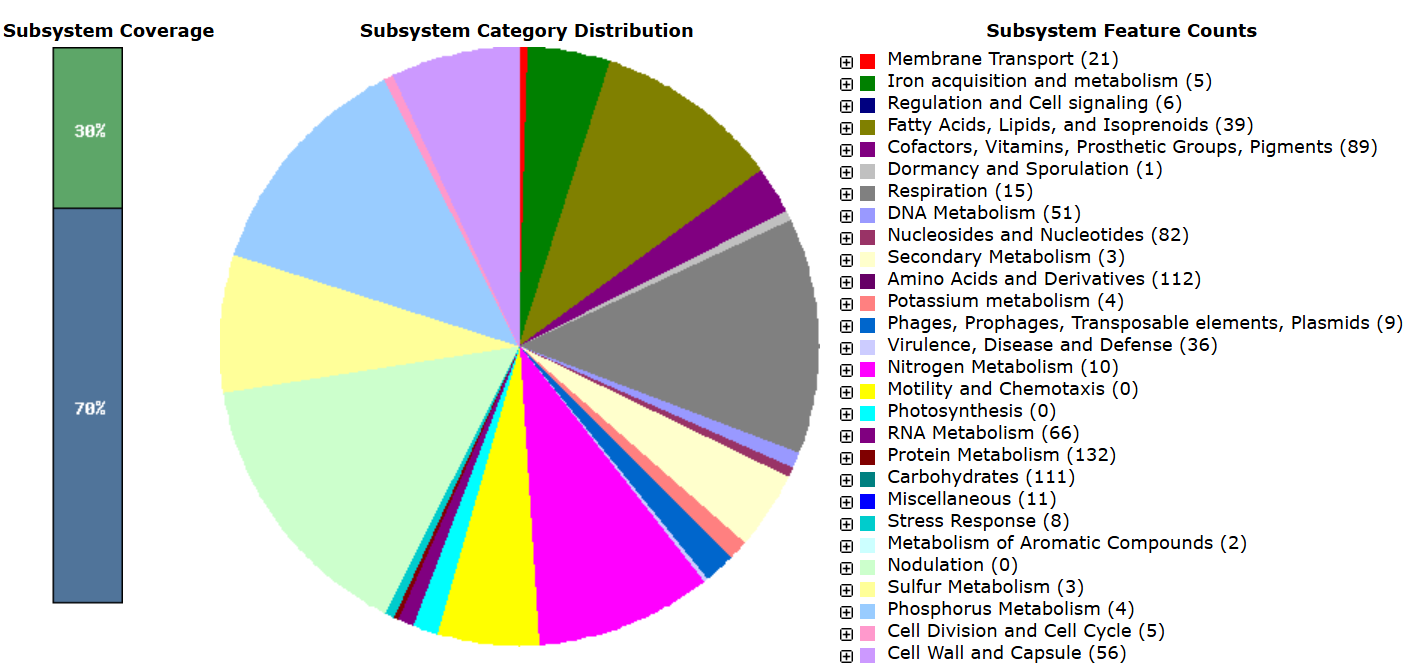
**

(Supplementary Fig. S7)

**
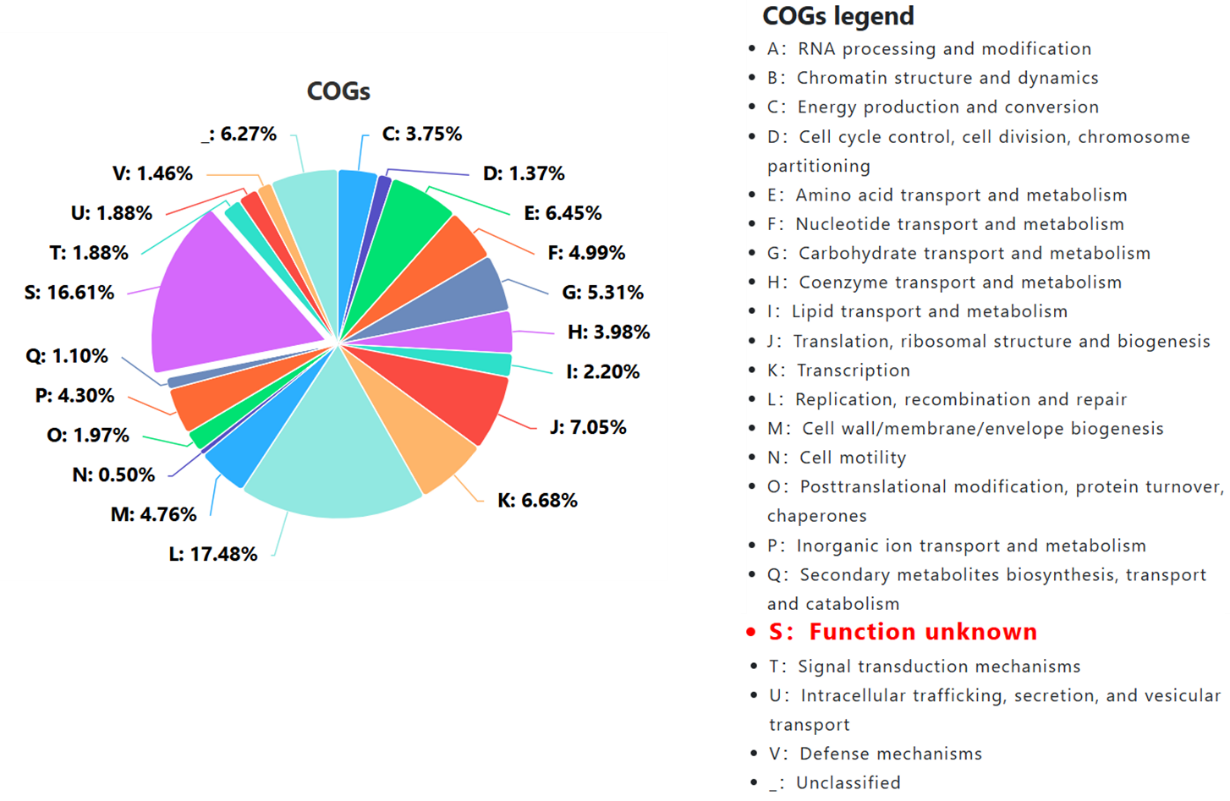
**

(Supplementary Fig. S8)


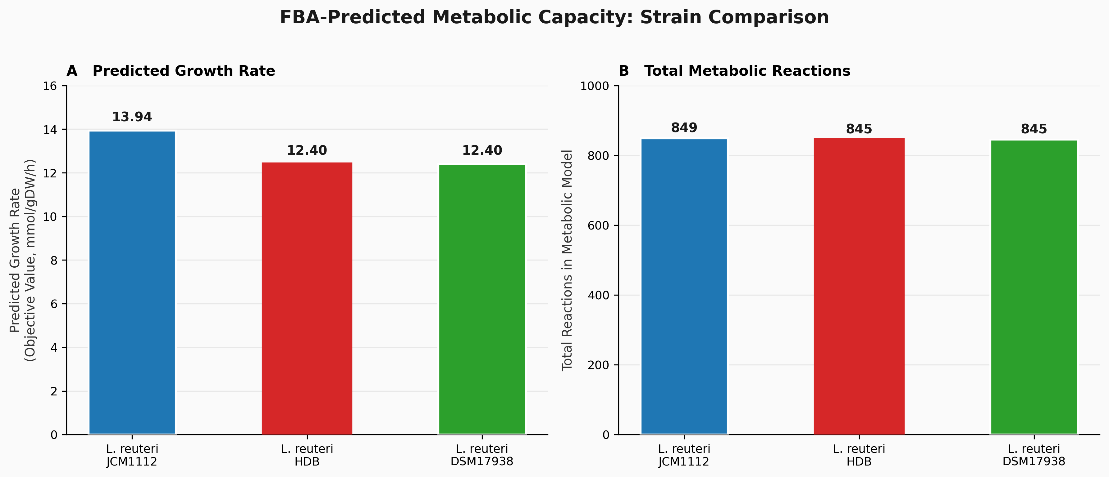


(Supplementary Fig. S9)


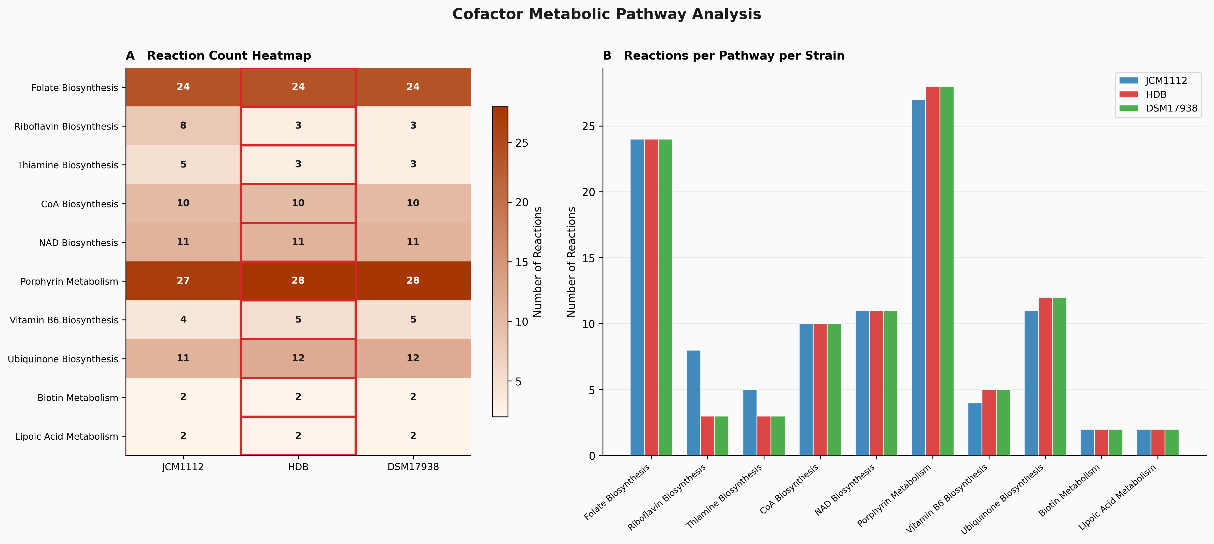


(Supplementary Fig. S10)


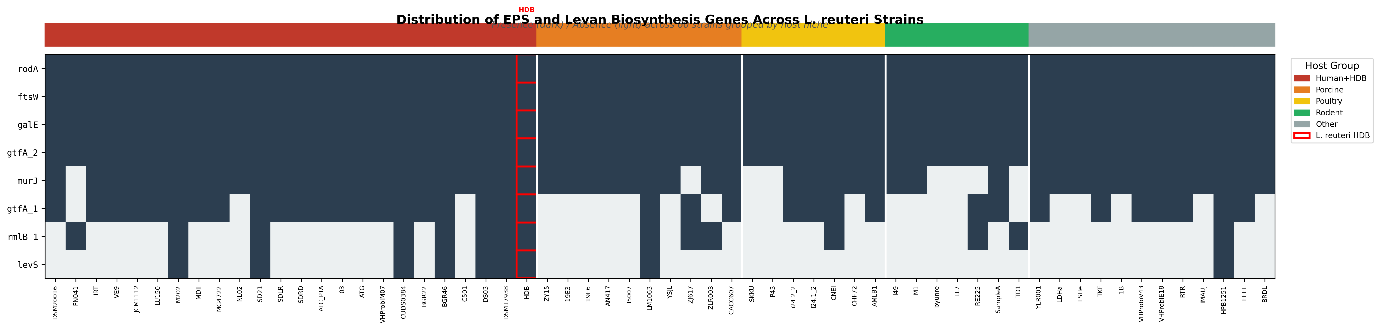


(Supplementary Fig. S11)

**
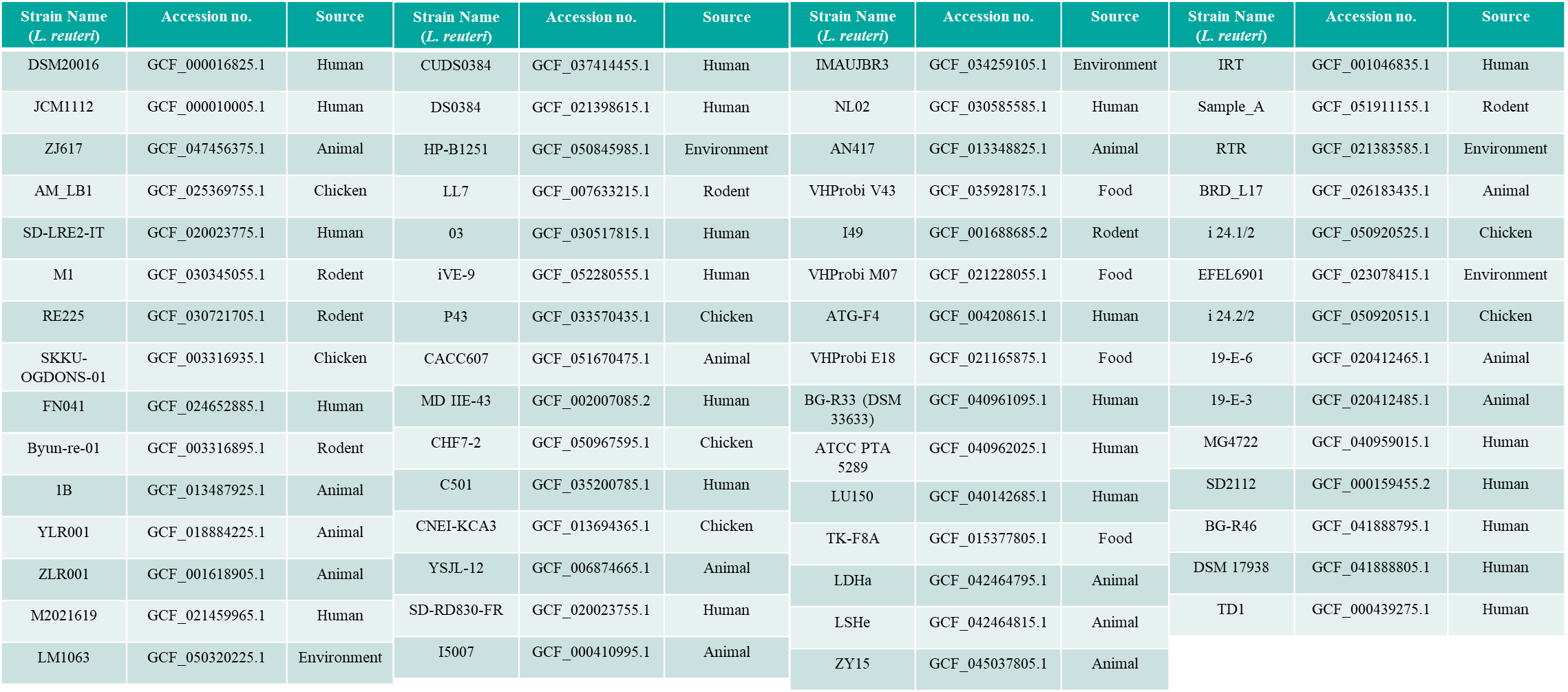
**

(Supplementary table 1)
